# The 14th Korea–Japan Joint Meeting for Vascular Surgery

**DOI:** 10.3400/avd.jk.26-01000

**Published:** 2026-05-23

Venue: Bexco Convention Hall 1F, Busan, Korea

Date: April 11 (Sat), 2026

KSVS President: Yong-Pil Cho, KSVS Chair of Board: Sang-Su Lee

## Free Paper Session I

**Moderators:**

Seung-Kee Min

Department of Surgery, Seoul National University College of Medicine, Seoul, Korea

Shinji Miyamoto

Department of Cardiovascular Surgery, Oita University, Yufu-shi, Japan

### O1-1 Early Outcomes of Endovascular Aneurysm Repair at a Newly Established Center

Hyung-jin Cho, Mi-hyeong Kim, Jisum Moon, Junsueng Kim, Jeong-kye Hwang

Division of Vascular and Transplant Surgery, Department of Surgery, Eunpyeong St. Mary’s Hospital, College of Medicine, The Catholic University of Korea, Seoul, Korea

#### INTRODUCTION

Clinical outcomes of endovascular aneurysm repair (EVAR) during the early adoption phase at newly established centers have been infrequently reported. This study aimed to evaluate early and mid-term outcomes of EVAR performed at a newly established hospital.

#### METHODS

Between July 2019 and January 2026, 112 EVAR procedures were performed. After excluding penetrating aortic ulcer and isolated iliac artery aneurysm, 78 patients with infrarenal abdominal aortic aneurysms were retrospectively analyzed. Baseline characteristics, aneurysm anatomy, perioperative variables, and follow-up outcomes were reviewed. Primary outcomes were all-cause mortality, abdominal aortic aneurysm (AAA)-related mortality, and re-intervention-free survival.

#### RESULT

The mean age was 77.2 ± 6.9 years, and 75.6% of patients were male. Complex aortic neck anatomy was present in 52.6% of cases. During a mean follow-up of 714 ± 648 days, Kaplan–Meier analysis demonstrated a 1-year overall survival of approximately 95% and a 1-year re-intervention-free survival of approximately 90%. AAA-related mortality remained low throughout follow-up.

#### CONCLUSION

EVAR performed during the early phase at a newly established center showed low AAA-related mortality and acceptable re-intervention rates, comparable to those reported by established centers.

**Figure:**
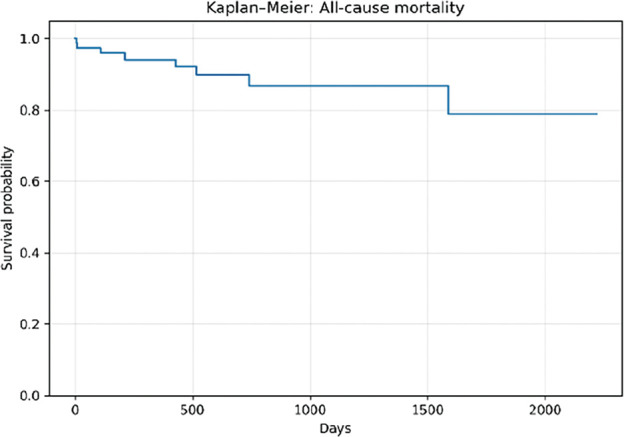


**Figure:**
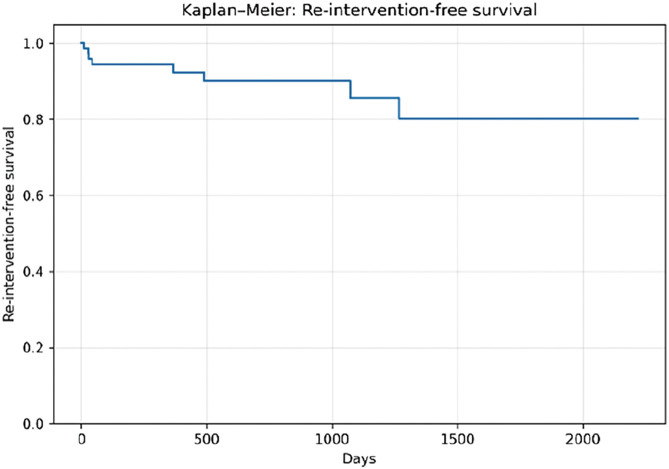


### O1-2 Assessments of Surgical Treatment for Superior Vena Cava (SVC)

Masayoshi Okada, Takaki Sugimoto, Hidehiro Yamamoto

Kobe University of Health, Japan

#### INTRODUCTION

In the recent years, the number of patients with superior vena cava (SVC) syndrome has been increasing in Japan. The causes of SVC syndrome include benign tumors, malignant tumors of the neck and thorax, such as lung cancer and Hodgkin lymphoma, and mediastinal tumors. These conditions can lead to symptoms such as dizziness and moon face. There are many types of surgical procedures for these patients.

#### METHODS

Among 24 patients (16 men, 8 women; ages 16–70 years, mean 49.1), clinical symptoms included edema and swelling of the head and neck. For these patients, we performed precise diagnoses using computed tomography, magnetic resonance imaging, and venography.

#### RESULT

We performed anatomic reconstructions in 19 patients and non-anatomic reconstructions in 5 patients after chemotherapy and roentgen therapy. After anastomotic reconstructions, all patients did well; among those who underwent non-anatomic reconstructions, only 1 patient survived 7 months without any complications.

#### CONCLUSION

1) Surgical reconstructions for SVC syndrome associated with lung cancer and mediastinal tumors can be effectively treated using ringed expanded polytetrafluoroethylene (e-PTFE), 2) As a graft prosthesis, we have used ringed e-PTFE, which proved sufficiently effective by double-bypass procedures. 3) In the recent years, stent insertion has emerged as a clinically effective alternative.

### O1-3 Vessel Belt: An Innovative Device for Flow Reduction in High-Flow Arteriovenous Fistula

Jun Yong Jekal, You Seok Chung, Kwang Hoon Je, Sang Su Lee

Pusan National University Yangsan Hospital, Korea

#### INTRODUCTION

High-flow access, commonly caused by progressive venous dilation after arteriovenous fistula (AVF) creation, can lead to excessive cardiac return and cardiac failure. We developed a novel device, the Vessel Belt, designed to encircle the vein, restrict diameter, and provide adjustable flow control. This study evaluated its feasibility, efficacy, and short-term safety in a porcine AVF model.

#### METHODS

The Vessel Belt consists of a flexible polyurethane band with ratchet projections and a metal locking wire, allowing adjustable circumferential tightening and stable fixation. AVFs were created between the deep femoral artery and vein in Yorkshire pigs. After 4–6 weeks, high-flow conditions were confirmed by Doppler ultrasound, and the Vessel Belt was applied to the common iliac vein. Vessel diameter and blood flow were measured before and immediately after application. The animals were maintained for 4 weeks, after which the treated venous segments were harvested for pathological evaluation.

#### RESULT

The device produced an immediate reduction in vessel diameter and blood flow, with stable handling and no migration during follow-up. Pathological examination was performed, and the results demonstrated the usability of the material and design.

#### CONCLUSION

The Vessel Belt effectively achieved controlled flow reduction in a porcine high-flow AVF (HFA) model with favorable short-term safety. This adjustable, externally applied device may provide a standardized treatment option for HFA. Further long-term and clinical studies are needed.

#### KEYWORDS

AVF, HFA, innovation, patent, invention

### O1-4 AI Model for Cross-Sectional Carotid Ultrasound Plaque Analysis

Jin Hyun Joh^1^, Hyunmo Yang^2^, Woonggyu Jung^2^

^1^Department of Surgery, Kyung Hee University Hospital at Gangdong, College of Medicine, Kyung Hee University, Seoul, Republic of Korea

^2^Department of Biomedical Engineering, College of Information and Biotechnology, Ulsan National Institute of Science and Technology (UNIST), Ulsan, Republic of Korea

#### INTRODUCTION

Carotid atherosclerosis is a primary factor in ischemic strokes and major cardiovascular events. While plaque presence and area provide critical risk information that exceeds traditional predictive factors, current quantification methods in ultrasound typically rely on manual tracing. These manual processes are often time-consuming and prone to operator variability, highlighting a clinical need for automated and reproducible analysis.

#### METHODS

To address this, a dual-model framework was developed using an Attention U-Net architecture, which utilizes attention gates to focus on relevant anatomical regions while ignoring background noise. The system consists of 2 separate deep-learning models for vessel compartment segmentation and plaque segmentation. The models were trained on 850 cross-sectional images from 301 patients, covering the common, internal, and external carotid arteries, as well as the carotid bulb.

#### RESULT

The performance of the models was validated against expert manual annotations using overlap metrics such as the Dice coefficient and distance-based measures. Results indicated that the pipeline produces anatomically plausible segmentations and enables the automated calculation of plaque-to-vessel area ratios—a quantitative surrogate for plaque burden. These models showed strong agreement with experts.

#### CONCLUSION

This artificial intelligence-based framework provides a rapid and objective method for measuring carotid plaque burden during routine clinical ultrasound.

### O1-5 Impact of Early Graft Patency on Survival after Bypass in Dialysis-Dependent CLTI

Keisuke Kamada, Shinsuke Kikuchi, Kyokei Fuchizawa, Izumi Fukii, Hirofumi Jinno, Takayuki Uramoto, Naoya Kuriyama, Nobuyoshi Azuma

Department of Vascular Surgery, Asahikawa Medical University, Asahikawa, Japan

#### INTRODUCTION

This study aimed to evaluate the impact of graft patency on long-term survival in hemodialysis-dependent (HD) patients with chronic limb–threatening ischemia (CLTI) undergoing infrainguinal bypass surgery.

#### METHODS

We retrospectively analyzed 304 HD patients with CLTI treated with bypass between 2000 and 2019. Patients were stratified into short- (<2 years) and long- (≥2 years) term survivors. Graft patency and survival outcomes were compared, and factors associated with early graft patency were assessed.

#### RESULT

Two- and 5-year survival rates were 52% and 25%, respectively. Long-term survivors showed significantly higher 2-year primary and secondary patency than short-term survivors (53% vs. 61%, P = 0.02; 79% vs. 88%, P = 0.04). Patency at 6 and 12 months was strongly associated with improved survival (both P <0.001). Higher WIfI (wound, ischemia, foot infection) wound grade predicted 6-month graft failure (wound grade ≥2; odds ratio [OR] 0.38), and higher foot infection grade predicted 12-month graft failure (foot infection grade ≥2; OR 0.33). Early graft patency independently predicted 2-year survival (6-month OR 3.72; 12-month OR 5.04).

#### CONCLUSION

Early graft patency after bypass surgery was strongly associated with improved long-term survival in HD patients with CLTI. Maintaining graft patency within the first postoperative year may be critical not only for limb salvage but also for overall survival. Strategies to optimize early graft durability could contribute to better long-term outcomes in this high-risk population.

#### KEYWORDS

hemodialysis, CLTI, bypass surgery, graft patency, survival

### O1-6 Robotic MALS Release and Neurolysis for Persistent Symptoms despite Patent Celiac Stenting

HaengJin OHE, Ki Yoon Moon, Eun Ju Jang, Jang Yong Kim, Sun Cheol Park, Sang Seob Yun

Seoul St. Mary’s Hospital, Korea

#### INTRODUCTION

Median arcuate ligament syndrome (MALS) involves compression of the extrinsic celiac trunk Endovascular stenting often fails to provide symptomatic relief, as it addresses neither the mechanical compression nor the celiac plexus involvement. We present a case of robotic-assisted release and neurolysis in a patient who remained symptomatic despite a patent celiac stent.

#### METHODS

A 41-year-old woman presented with classic postprandial pain and weight loss. She had a prior celiac stent placed elsewhere. Although Doppler ultrasound confirmed a patent stent, her symptoms persisted. We performed robotic-assisted surgery using the da Vinci system. Meticulous dissection was conducted to divide the median arcuate ligament and perform extensive celiac plexus neurolysis.

#### RESULT

The robotic platform enabled safe dissection around the pre-existing stent. There were no complications. Postoperatively, the patient reported complete symptom resolution and resumed a regular diet. This demonstrates that her pain was driven by extrinsic mechanical and neurogenic factors rather than luminal narrowing alone.

#### CONCLUSION

Technical stenting success does not guarantee clinical improvement in MALS. Robotic surgery offers a superior, minimally invasive approach for definitive decompression and neurolysis, even after prior endovascular intervention.

### O1-7 Clinical Outcomes and Treatment Strategies in Korean Visceral Artery Aneurysms

Jayeon Ahn, Sanghyun Ahn, Sangil Min, Jongwon Ha, Ara Cho, Seung-Kee Min

Seoul National University College of Medicine, Korea

#### INTRODUCTION

Visceral artery aneurysms (VAA) are rare and their natural history and prognosis in Koreans are not well understood. This study aimed to analyze VAA characteristics, treatment outcomes, rupture rates, and risk factors in Korean patients as part of the K-RARE-03 project.

#### METHODS

A retrospective study of 510 VAA lesions diagnosed at Seoul National University Hospital (2004–2024) analyzed 77 lesions (excluding splenic and renal aneurysms) by location, rupture risk, growth rate, and surgical indications.

#### RESULT

VAA occurrence rates: SMA (4.1%), hepatic (3.7%), celiac (3.1%), pancreatic (2.7%), GD (0.6%), IMA (0.2%). Most aneurysms were incidentally found, and 70% were of unknown origin. Thirty-eight percent of SMA aneurysms were due to dissection. Fusiform aneurysms predominated in SMA/celiac arteries, while saccular aneurysms were more common elsewhere. Seventy-one percent of pancreatic aneurysms had celiac axis occlusion. Three ruptured aneurysms were identified: 2 hepatic and 1 right colic; all were first discovered as ruptures in patients with Ehlers–Danlos syndrome and polyarteritis nodosa. No deaths were reported. Growth rates were slow: hepatic 1.77 mm/year, SMA 0.06 mm/year, celiac 0.18 mm/year, and pancreatic 0.34 mm/year. Treatment was mainly based on location and size, with 50% of pancreatic, 42% of hepatic, 33% of gastroduodenal artery, and 19% of SMA aneurysms treated within 1 year.

#### CONCLUSION

Most aneurysms showed minimal growth, and no ruptures occurred in patients without underlying comorbidities. Further studies are needed for standardized VAA guidelines.

### O1-8 Elective EVAR in Nonagenarians: Anatomical Features and Late Complications Compared to Octogenarians

Hideyuki Hayashi, MD^1^, Naoki Fujimura, MD, PhD^1^, Hideaki Obara, MD, PhD^1^, Tatsuya Shimogawara, MD^2^, Shigeshi Ono, MD, PhD^3^, Keita Hayashi, MD^4^, Susumu Watada, MD, PhD^5^, Taku Fujii, MD, PhD^6^, Yasuhito Sekimoto, MD, PhD^7^, Tsunehiro Shintani, MD, PhD^8^, Hirohisa Harada, MD, PhD^9^, Norio Uchida, MD, PhD^10^, and Yuko Kitagawa, MD, PhD^1^

^1^Department of Surgery, Keio University School of Medicine, Tokyo, Japan

^2^Department of Vascular Surgery, Saiseikai Yokohamashi Tobu Hospital, Kanagawa, Japan

^3^Department of Surgery, Tokyo Dental College Ichikawa General Hospital, Chiba, Japan

^4^Department of Vascular Surgery, Hiratsuka City Hospital, Kanagawa, Japan

^5^Department of Surgery, Kawasaki Municipal Hospital, Kanagawa, Japan

^6^Department of Surgery, Saitama Municipal Hospital, Saitama, Japan

^7^Department of Surgery, National Hospital Organization Tokyo Medical Center, Tokyo, Japan

^8^Department of Vascular Surgery, Shizuoka Red Cross Hospital, Shizuoka, Japan

^9^Department of Vascular Surgery, Saiseikai Central Hospital, Tokyo, Japan

^10^Department of Surgery, Mito Red Cross Hospital, Ibaraki, Japan

#### INTRODUCTION

Previous nationwide studies have reported comparable early outcomes after endovascular aneurysm repair (EVAR) between nonagenarians and octogenarians, but detailed anatomical features and late complications have not been investigated. This study aimed to evaluate these aspects in Japanese patients undergoing elective EVAR.

#### METHODS

A retrospective analysis was performed using a prospectively maintained multicenter EVAR registry (July 2007–August 2024). Cases with ruptured, inflammatory, or infected aneurysms and complex EVAR were excluded.

#### RESULT

Of 2677 eligible cases, 84 were nonagenarians (92.6 ± 2.3 years) and 953 were octogenarians (83.2 ± 3.3 years). Nonagenarians had larger aneurysms (55.9 vs. 51.0 mm, P <0.001), more ASA >3 (42.9% vs. 27.8%, P = 0.004), and greater neck angulation (56.8° vs. 41.4°, P <0.001), leading to higher IFU (instructions for use) violations (70.2% vs. 55.4%, P = 0.009). They showed more type II endoleaks (45.2% vs. 32.7%, P = 0.020) and less sac regression (22.6% vs. 35.3%, P = 0.019), but 30-day adverse events, 3-year mortality, and 5-year freedom from late complications were comparable.

#### CONCLUSION

Despite more complex anatomy, elective EVAR in nonagenarians achieved mid-term outcomes similar to those in octogenarians. Age ≥90 years alone should not be considered a contraindication for EVAR in appropriately selected patients.

**Figure:**
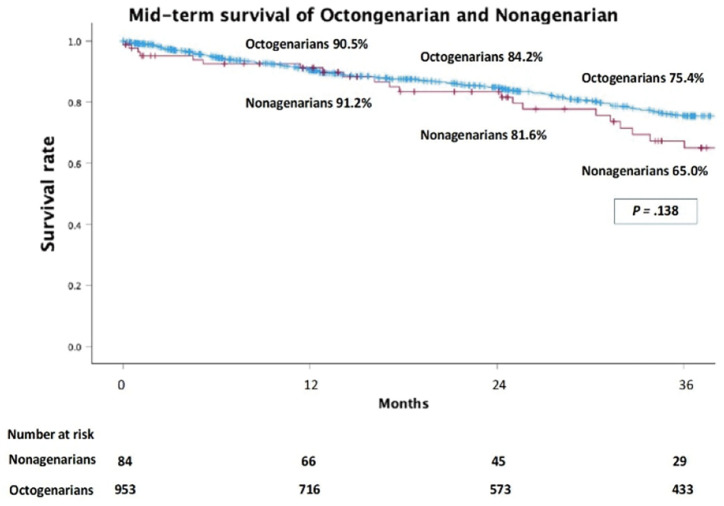


**Figure:**
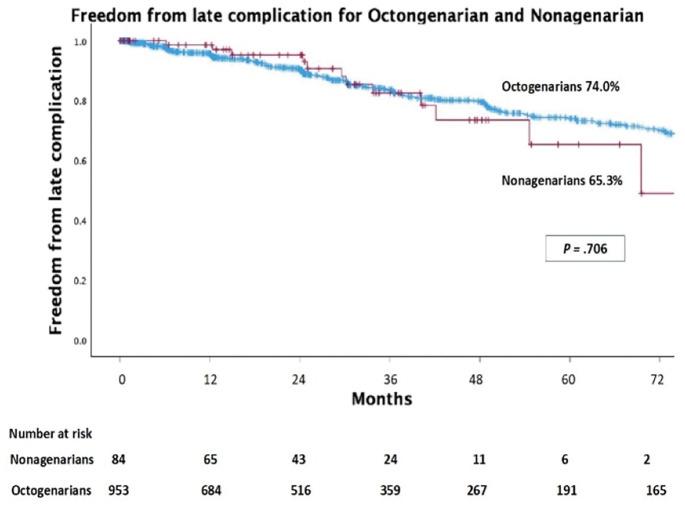


### O1-9 Surgical Management of MALS-Related Pancreaticoduodenal Artery Aneurysms

Hirona Todoroki, Masafumi Chikamatsu, Takuya Osawa, Changi Lee, Shuta Ikeda, Naohiro Akita, Masayuki Sugimoto, Hiroshi Banno

Division of Vascular and Endovascular Surgery, Department of Surgery, Nagoya University Graduate School of Medicine, Aichi, Japan

#### OBJECTIVE

Celiac artery occlusion due to median arcuate ligament syndrome (MALS) increases collateral blood flow from the superior mesenteric artery (SMA) to the pancreaticoduodenal artery (PDA), leading to the formation of PDA aneurysms (PDAA). While treatment is recommended regardless of aneurysm size, the optimal strategy for MALS-related PDAA remains controversial. This study aimed to evaluate the outcomes of open surgical management for unruptured PDAA associated with MALS.

#### METHODS

This single-center retrospective study reviewed 11 patients who underwent surgical treatment for unruptured MALS-related PDAA between April 2016 and July 2025. Open surgery was selected over endovascular treatment in all cases to ensure preservation of hepatic blood flow and due to the anatomical proximity of the aneurysms to the SMA.

#### RESULTS

There was no surgical mortality. Postoperative complications included graft occlusion without organ ischemia in 1 patient, delayed gastric emptying (DGE) in 5, and pancreatic fistula in 2. During the follow-up period, no aneurysm recurrence or late bypass occlusion was observed. Among the 6 patients followed for over 1 year, symptoms persisted in 2 (1 with diarrhea and 1 with DGE). Notably, 1 small untreated aneurysm demonstrated shrinkage postoperatively.

#### CONCLUSION

Open surgical repair for MALS-related PDAA is safe and effective in preventing rupture and recurrence. Although DGE is a frequent postoperative complication, restoring antegrade celiac flow is crucial, as it may promote the regression of residual aneurysms and prevent hepatic ischemia.

## Free Paper Session II

**Moderators:**

Sun Cheol Park

Division of Vascular Surgery, Department of Surgery, Seoul St. Mary's Hospital, The Catholic University of Korea, Seoul, Korea

Hiroyoshi Komai

Department of Vascular Surgery, Kansai Medical University Medical Center, Osaka, Japan

### O2-1 Assisted Maturation Does Not Compromise Long-Term Patency of Native Arteriovenous Fistula

Sangmin Gong, Youngjin Han

Division of Vascular Surgery, Department of Surgery, College of Medicine, University of Ulsan, Asan Medical Center, Seoul, Korea

#### INTRODUCTION

Whether assisted maturation (AM), defined as intervention within 90 days of native arteriovenous fistula (AVF) creation, affects long-term patency remains unclear.

#### METHODS

We analyzed 379 native AVFs created between March 2023 and February 2025 at a single center, followed through January 2026. Kaplan–Meier analysis was used to estimate unassisted primary patency (UPP), assisted primary patency (APP), and secondary patency (SP). APP and SP were compared between the AM and non-AM groups using log-rank tests and multivariable Cox regression, adjusting for age, sex, dialysis status, diabetes, coronary artery disease, and AVF location.

#### RESULT

The median age was 65 years (interquartile range 54–73); 61.7% were male. Thirty-eight AVFs (10.0%) required AM, including balloon-assisted maturation, percutaneous transluminal angioplasty, or surgical revision. The 12- and 24-month patency rates were 76.0% and 72.8% for UPP, 91.6% and 88.7% for APP, and 92.7% and 91.3% for SP. The AM group had higher rates of ongoing hemodialysis (68.4% vs. 46.6%, p = 0.017) and coronary artery disease (31.6% vs. 12.0%, p = 0.002). Despite this, no significant differences were found in APP (p = 0.422) or SP (p = 0.953). On multivariable Cox regression, AM was not associated with APP (hazard ratio [HR] 1.605, 95% confidence interval [CI] 0.599–4.304, p = 0.347) or SP (HR 1.018, 95% CI 0.296–3.501, p = 0.978).

#### CONCLUSION

Native AVFs showed favorable long-term patency. AM did not compromise long-term outcomes, suggesting that AVFs requiring early intervention can achieve durable function with appropriate management.

#### KEYWORDS

arteriovenous fistula, vascular patency, hemodialysis, vascular access maturation, endovascular procedures

**Figure:**
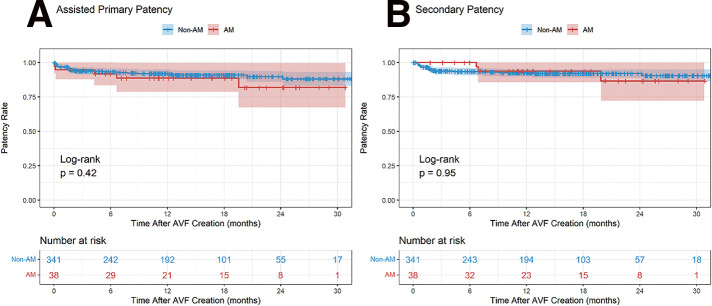


**Figure:**
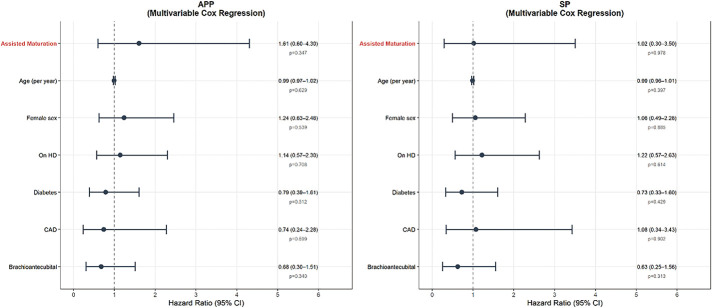


### O2-2 Analysis of Optimal Hemoglobin Threshold for Preventing Myocardial Injury after AAA Open Repair

Hyunjung Ryu

Samsung Medical Center, Korea

#### INTRODUCTION

Myocardial injury after non-cardiac surgery (MINS) significantly impacts outcomes after abdominal aortic aneurysm (AAA) open repair. Postoperative anemia is a known risk factor, but the optimal hemoglobin (Hb) threshold for MINS remains unclear. This study aims to identify the nadir Hb level predictive of MINS and its effect on long-term survival.

#### METHODS

We retrospectively analyzed 328 patients undergoing elective infrarenal AAA open repair at a single center. MINS was defined as isolated cardiac troponin elevation within postoperative days 0–3. The optimal nadir Hb threshold was determined via receiver operating characteristic (ROC) curve analysis. Multivariable logistic regression was performed to identify independent risk factors.

#### RESULT

The MINS(+) group had significantly lower nadir Hb levels (8.7 vs. 9.7 g/dL, p <0.001). ROC analysis identified 9.45 g/dL as the optimal threshold (area under the curve 0.68). In multivariable analysis, nadir Hb <9.45 g/dL was a potent independent predictor of MINS (adjusted odds ratio 5.183; 95% confidence interval CI 1.670–16.082; p = 0.004). Patients with MINS demonstrated significantly higher 30-day major adverse cardiovascular events (p <0.001) and reduced 5-year overall survival (p = 0.022).

#### CONCLUSION

Postoperative nadir Hb <9.45 g/dL is associated with a 5-fold increase in MINS risk and poorer long-term survival after infrarenal AAA open repair. This threshold is higher than conventional restrictive transfusion triggers (7.0–8.0 g/dL). Our findings suggest that even in elective infrarenal cases, more aggressive anemia management may be required to mitigate myocardial risk and improve clinical outcomes.

**Figure:**
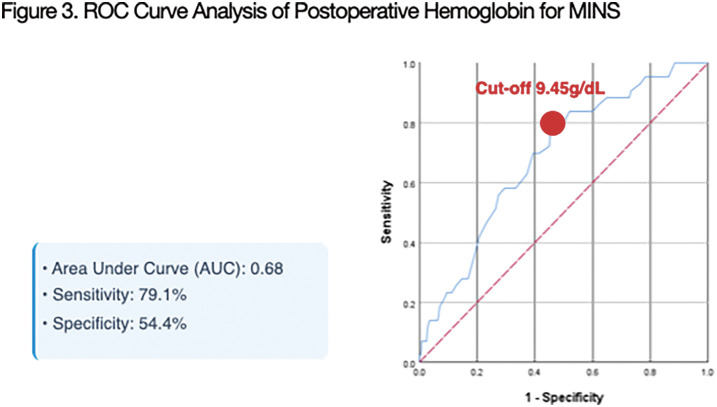


**Figure:**
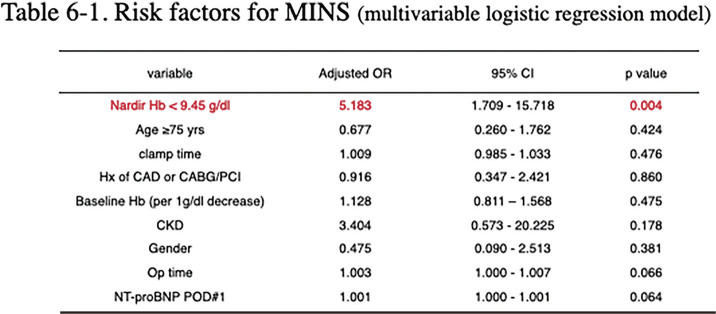


### O2-3 Operative Time in Distal Bypass: Impact on Mid-Term Outcomes and Predictors

Taku Kokubo, Tadahiro Sasajima

Department of Vascular Surgery, Sapporo Kojinkai Memorial Hospital, Japan

#### INTRODUCTION

This study aimed to evaluate the impact of operative time on mid-term clinical outcomes following distal bypass and to identify independent predictors of prolonged surgical duration.

#### METHODS

We retrospectively reviewed 879 infrapopliteal bypasses performed on 663 patients between 2012 and 2025. The study cohort had a mean age of 70 years and high rates of comorbidities, including diabetes mellitus (78%), ischemic heart disease (49%), and hemodialysis (47%).

#### RESULT

Multivariate analysis demonstrated that each 10-min increment in operative time was significantly associated with an increased risk of 1-year primary patency loss (odds ratio [OR], 1.04; 95% confidence interval [CI], 1.03–1.06; P <0.001) and major amputation (OR, 1.04; 95% CI, 1.02–1.06; P <0.001). Increased operative time was also a significant predictor of 2-year mortality (OR, 1.02; 95% CI, 1.01–1.04; P = 0.007). Independent predictors of prolonged operative duration included concomitant inflow reconstruction (P <0.01), non-single-piece vein grafts (P <0.001), poor runoff (P <0.001), and the peroneal artery as the distal target (P <0.001).

#### CONCLUSION

Prolonged operative time is a potent negative prognostic factor for graft patency, limb salvage, and survival in distal bypass. In complex cases involving suboptimal grafts or challenging anatomical targets, meticulous preoperative planning and technical optimization to minimize surgical duration are crucial for improving clinical outcomes.

#### KEYWORDS

distal bypass, operative time, mid-outcomes, limb salvage

### O2-4 Hemodynamic Characteristics of the Posteromedial Wall at the Common Femoral Artery Bifurcation

Kiyoon Moon, Eunju Jang1, Hang Jin Oh, Sangseob Yun, Suncheol Park, Jangyong Kim

Division of Vascular and Transplant Surgery, Department of Surgery, The Catholic University of Korea, Korea

#### INTRODUCTION

Atherosclerotic involvement of the common femoral artery (CFA) often shows preferential localization along the posterior and medial arterial wall. The local hemodynamic characteristics associated with this spatial predilection remain incompletely defined. This study aimed to characterize patient-specific flow patterns and wall shear metrics at the CFA bifurcation using computational fluid dynamics (CFD).

#### METHODS

A 3-dimensional aortofemoral arterial model was reconstructed from contrast-enhanced computed tomography images. Pulsatile blood flow simulation was performed using SimVascular. Time-resolved velocity vector fields were analyzed at the common femoral artery (CFA) bifurcation during peak systole, systolic deceleration, and end diastole. Spatial distributions of velocity magnitude, time-averaged wall shear stress (TAWSS), and oscillatory shear index (OSI) were evaluated.

#### RESULT

Flow visualization demonstrated asymmetric velocity profiles and secondary flow patterns at the CFA bifurcation. Flow disturbance and low-velocity regions were most pronounced along the posteromedial CFA during systolic deceleration and early diastole. Reduced TAWSS and elevated OSI were consistently observed in the posteromedial region compared with other wall segments.

#### CONCLUSION

Patient-specific CFD analysis revealed spatially heterogeneous hemodynamic features at the CFA bifurcation, characterized by disturbed flow, reduced wall shear stress, and increased oscillatory shear along the posteromedial arterial wall.

**Figure:**
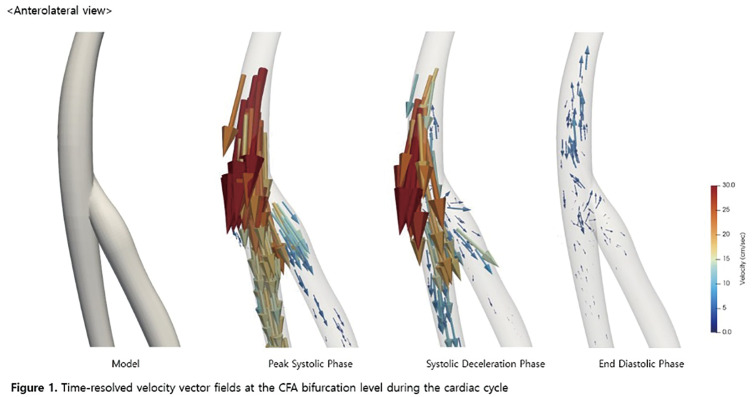


**Figure:**
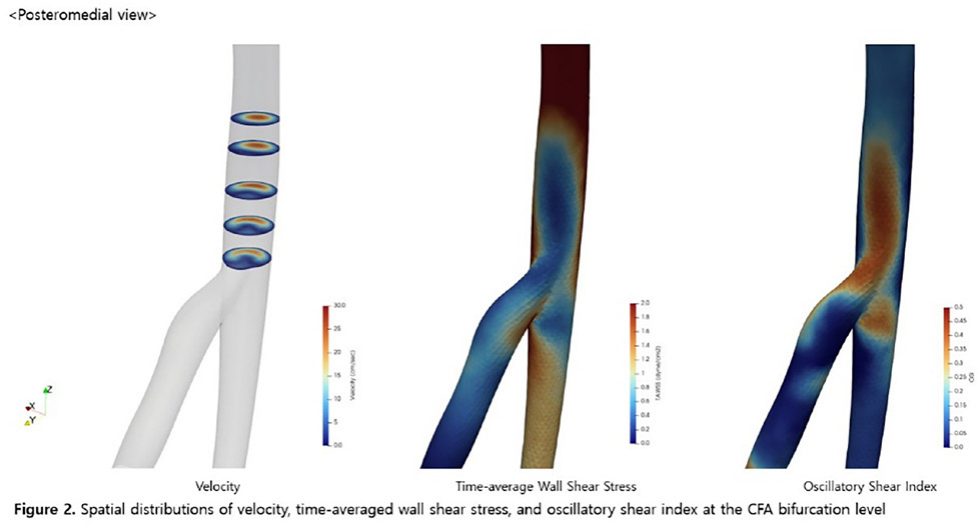


### O2-5 The Fate of the Wound Beyond the Angiosome: Role of the Collateral Bridge and Pedal Arch in CLTI

Hyo Jun Kim, Hyung Sub Park, Jinsol Jung, Jonghyuk Park, Jin Myung Yoon, Taeseung Lee

Division of Vascular Surgery, Department of Surgery, Seoul National University Bundang Hospital, Korea

#### INTRODUCTION

Angiosome-directed revascularization has traditionally guided target artery selection in chronic limb–threatening ischemia (CLTI), yet wound healing after indirect revascularization remains variable. We evaluated outcomes according to wound–target artery relationship (WTR), incorporating collateral connectivity and pedal arch integrity to better define functional perfusion to the wound.

#### METHODS

We retrospectively analyzed 146 limbs in 133 CLTI patients with foot wounds undergoing revascularization (2014–2021). WTR was classified as direct (Status 1), indirect with functional collaterals (Status 2), and indirect without collaterals (Status 3). Pedal anatomy was assessed using Kawarada classification and GLASS (Global Limb Anatomic Staging System) pedal modifier. Primary outcomes were complete wound healing and limb salvage.

#### RESULT

Healing was comparable between Status 1 and Status 2 (54.9% vs. 59.2%), with none in Status 3 (p <0.001). Healing declined stepwise by pedal status: 72.2% (T1), 55.8% (T2), and 0% (T3), with a similar gradient by modifier: 76.5% (P0), 54.2% (P1), and 0% (P2). Limb salvage exceeded 95% in T1–T2 but fell to 68.8% in T3. Kawarada class independently predicted healing in the multivariable Cox analysis, and collateral absence increased reintervention.

#### CONCLUSION

Wound healing in CLTI is closely associated with collateral connectivity and pedal arch integrity. Indirect revascularization via functional collaterals achieved outcomes comparable to direct flow, whereas advanced pedal outflow disease predicted nonhealing and reduced limb salvage.

#### KEYWORDS

chronic limb–threatening ischemia, limb salvage, angioplasty

**Figure:**
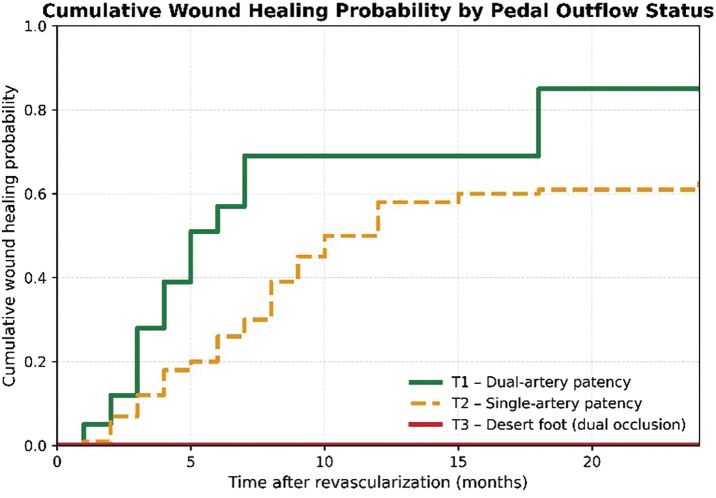


### O2-6 Early Outcomes of the Alto Abdominal Stent Graft System at Our Institution

Y. Aoyagi, K. Hashizume, D. Harada, M. Kasai, M. Mori, M. Nishida, H. Shimizu

Department of Cardiovascular Surgery, Saiseikai Utsunomiya Hospital, Japan

#### INTRODUCTION

The Alto Abdominal Stent Graft System, introduced in May 2022, features a biocompatible polymer-filled sealing ring that conforms to various proximal neck morphologies and may be suitable for hostile neck anatomies in abdominal aortic aneurysm (AAA). We report the early outcomes of endovascular aneurysm repair (EVAR) using this device.

#### METHODS

We retrospectively reviewed 16 patients who underwent EVAR with the Alto Abdominal Stent Graft System between May 2022 and August 2025.

#### RESULT

The male-to-female ratio was 14:2, and the mean age was 77 ± 8.9 years. There was no perioperative mortality. The mean follow-up period was 24 ± 15.4 months. Seven patients had short necks (proximal landing length <15 mm), 3 had calcified proximal necks, and 6 had reverse-taper necks, defined as an increase in aortic diameter of 2 mm or more at 10 mm distal to the proximal neck. The mean proximal landing length was 18 mm. There were no access-related complications. Final angiography showed no type Ia endoleak, while 4 patients had type II endoleaks. Postoperative computed tomography demonstrated type II endoleaks; however, during follow-up, no aneurysm sac enlargement, stent migration, reintervention, or mortality was observed.

#### CONCLUSION

Early outcomes of EVAR using the Alto endograft for AAA with hostile neck anatomy were favorable, with only type II endoleaks and no aneurysm growth. Although long-term durability requires further evaluation, the Alto endograft may be a useful option for challenging proximal neck anatomy.

#### KEWORDS

EVAR, AAA, Alto, hostile neck

**Figure:**
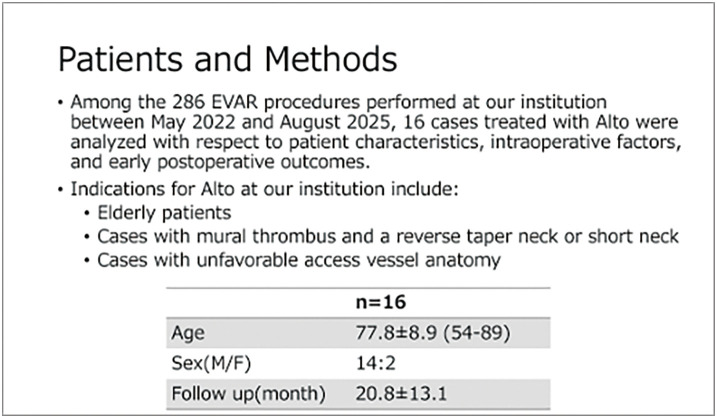


**Figure:**
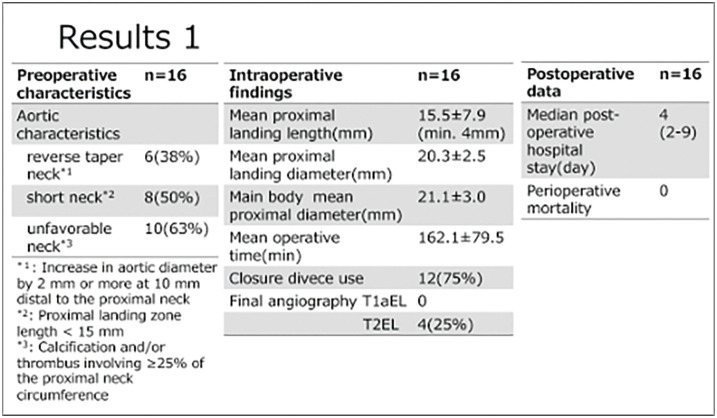


**Figure:**
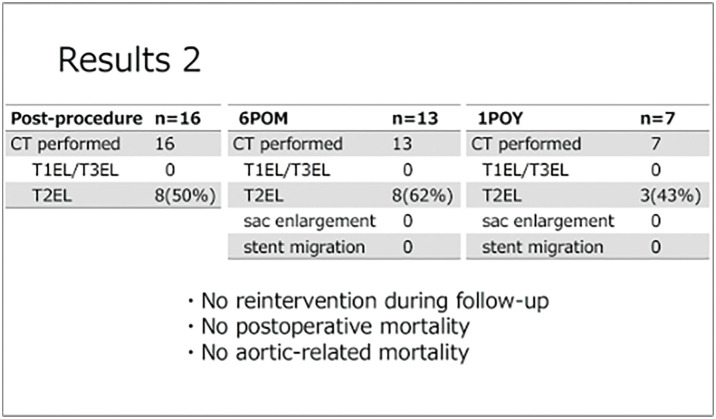


### O2-7 Partial versus Total Graft Excision for Localized Arteriovenous Graft Infection

Kee-Hyun Park, Deokbi Hwang, Hyung-Kee Kim, and Seung Huh

Division of Vascular and Endovascular Surgery, Department of Surgery, Kyungpook National University Hospital, Kyungpook National University School of Medicine, Daegu, South Korea

#### INTRODUCTION

Infection of prosthetic arteriovenous grafts (AVGs) is a common complication of hemodialysis access and may lead to serious morbidity. Surgical management requires balancing infection eradication with preservation of dialysis access.

#### METHODS

We retrospectively reviewed patients who underwent excisional surgery for prosthetic AVG infection between 2016 and 2025 at 2 tertiary referral centers. A total of 86 patients were analyzed: total graft excision (TGE, n = 13) and partial graft excision (PGE, n = 73). Outcomes were recurrent infection within 1 month after discharge and 30-day mortality.

#### RESULT

Recurrent infection rates were not associated with arterial anastomosis involvement, wound closure strategy, or excision type (TGE vs. PGE). Among patients with bacteremia (n = 25), PGE was not associated with a higher risk of recurrent infection compared with TGE. Thirty-day mortality occurred in 2 patients in the PGE group. Notably, 11 infections involved nonfunctional residual grafts.

#### CONCLUSION

PGE was not associated with higher rates of recurrent infection compared with TGE in patients with localized AVG infection, even in the presence of bacteremia. Routine total graft excision may therefore not be necessary in selected patients. However, given the substantial risk of infection associated with nonfunctional residual grafts, removal of abandoned graft material should be considered.

### O2-8 Anatomical and Operator-Related Factors Associated with Technical Failure of Perclose™

Hirofumi Jinno, Shinsuke Kikuchi, Tsutomu Doita, Izumi Fukii, Kyokei Fuchizawa, Takayuki Uramoto`, Seima Ohira, Keisuke Kamada, Naoya Kuriyama, Nobuyoshi Azuma

Department of Vascular Surgery, Asahikawa Medical University, Asahikawa, Hokkaido, Japan

#### INTRODUCTION

Perclose™ is widely used as a vascular closure device for large-bore sheaths. However, device failure and access-site complications remain clinically relevant problems. We aimed to identify anatomical and operator-related factors associated with technical failure and to clarify when alternative strategies should be considered.

#### METHODS

We conducted a single-center retrospective study of patients who underwent endovascular aortic repair or endovascular therapy using Perclose™ at the common femoral artery (CFA) from 2020 to 2025. Preoperative computed tomography was used to evaluate CFA diameter, puncture depth, and calcification score (0–4). Operator factors included experience and cumulative device use. Technical failure was defined as unsuccessful device deployment or access-site complications. Multivariate logistic regression was performed to identify independent predictors.

#### RESULT

A total of 300 patients (485 limbs) were analyzed. Technical failure occurred in 52 limbs, and 75% of failures required surgical repair. Independent predictors included operator experience ≤10 cases (odds ratio [OR]: 5.469; p = 0.023), CFA diameter <8.0 mm (OR: 0.488; p = 0.022), puncture depth <15.35 mm (OR: 2.422; p = 0.013), and calcification score >3 (OR: 4.891; p = 0.014).

#### CONCLUSION

Technical failure of Perclose™ was associated with unfavorable puncture site anatomy and limited operator experience. Careful imaging assessment and awareness of the learning curve are important. In high-risk patients, alternative closure or cutdown should be considered.

## Poster Presentation I

**Moderators:**

Soo Jin Na Choi

Division of Transplantation and Vascular Surgery, Chonnam National University Hospital, Gwangju, Korea

Katsuyuki Hoshina

Department of Vascular Surgery, The University of Tokyo, Japan

### P1-1 Narrative Review of Venous Thrombosis During Aerospace Flight, 4th Report

Takaya Murayama, Kazuyo Sujino, Mitsumi Yamashita

Kannai Medical Clinic, Japan

#### INTRODUCTION

One of the serious complications during long-haul commercial flights is thromboembolism. Also, venous thrombosis during spaceflight has been a major problem since 2019, when asymptomatic thrombosis was reported in the left internal jugular vein of healthy astronauts.

#### METHODS

Studies from PubMed and Jstage were selected from 2000 to 2025. This review is not comprehensive or systematic but rather focuses on the most important aspects.

#### RESULT

Strategies to prevent venous thrombosis during commercial aviation flights include pharmacological prophylaxis for high-risk individuals and non-pharmacological therapies such as compression stockings, with risk stratification and stage-tailored prophylaxis recommendations. In weightlessness, the blood flow dynamics of the astronaut's internal jugular vein change, and thrombosis in the left internal jugular vein is a problem, but the exact cause and prevention method are still unknown, and research continues. In addition, in the increasingly active commercial spaceflight, the nonprofessional astronaut with a medical history also has more opportunities to fly in space. It is important to consider whether risk stratification measures in commercial aviation can be modified and applied to space flights as well.

#### CONCLUSION

Changes in the situation are accelerating, such as commercial spaceflights, the Artemis program, and exploration of Mars. There are many research issues that we venous lymph specialists should conduct.

#### KEYWORDS

spaceflight, aviation, venous thrombosis, review

### P1-2 Preoperative Endoleak Classification vs. Intraoperative Findings in Open Conversion after EVAR

Joon-Kee Park, MD^1^, Yang-Jin Park, MD, PhD^1^, Min-Hyung Lee, MD^1^, Hyun-Jung Ryu, MD^1^, Sang-Yup Lee, MD^2^, Shin-Seok Yang, MD^1^, and Young-Wook Kim, MD, PhD^3^

^1^Division of Vascular Surgery, Department of Surgery, Samsung Medical Center, Sungkyunkwan University School of Medicine, Seoul, Korea

^2^Department of Radiology, Samsung Medical Center, Sungkyunkwan University School of Medicine, Seoul, Korea

^3^Division of Vascular Surgery, Department of Surgery, Incheon Sejong Hospital, Incheon, Korea

#### INTRODUCTION

The diagnostic performance of preoperative imaging for identifying true high-pressure endoleaks prior to open surgical conversion (OSC) remains uncertain. This study aimed to evaluate discrepancies between preoperative endoleak classification and intraoperative findings in patients undergoing OSC.

#### METHODS

We conducted a retrospective single-center cohort study of patients who underwent OSC between 2009 and 2025. Preoperative duplex ultrasound (DUS), computed tomography angiography (CTA), and diagnostic angiography performed within 6 months were assessed. The reference standard was direct intraoperative confirmation of high-pressure endoleaks, defined as Type I or Type III. Diagnostic sensitivity, specificity, and overall accuracy, were evaluated.

#### RESULT

A total of 94 patients were included, with a median interval of 69 months from endovascular aneurysm repair (EVAR) to OSC. For type Ib endoleaks, sensitivity was 40.0% for DUS and 33.3% for CTA. Diagnostic performance was even poorer for type III endoleaks, with sensitivities of only 14.7% for DUS and 42.1% for CT angiography. Type III endoleaks showed high rates of concordant nondetection, with 41.7% of cases being missed by both DUS and CTA.

#### CONCLUSION

Preoperative imaging significantly underestimates the presence of intraoperative high-pressure endoleaks in patients with post-EVAR sac enlargement, particularly for Type III failures. Persistent sac enlargement warrants close surveillance even in the absence of imaging evidence of a high-pressure endoleak.

### P1-3 A Bypass Case for Buerger’s Disease after Autologous Bone Marrow Mononuclear Cell Transplantation

Noriyuki Miyama, Nobuko Yamamoto, Masato Ohno, Hiroyoshi Komai

Department of Vascular Surgery, Kansai Medical University Medical Center, Japan

#### INTRODUCTION

The patient was a 38-year-old man who first presented with intermittent claudication at age 32 and was diagnosed with Buerger’s disease. At age 34, he developed an ulcer on his left first toe and underwent autologous bone marrow mononuclear cell transplantation at another institution, which successfully healed the ulcer. Although he had not smoked since then, necrosis was observed in the left first toe at age 38. The necrosis progressed rapidly despite conservative treatment, leading to his transfer to our department.

#### METHODS

Angiography revealed occlusion from the distal superficial femoral artery (SFA) to the infrapopliteal artery. While the proximal portion of anterior tibial artery and tibioperoneal (PT) trunk were visualized, all major arteries in the mid-to-distal tibial regions were occluded. Numerous collateral vessels were observed in these regions, and the presence of small plantar arteries was confirmed.

#### RESULT

A bypass surgery was performed from the mid-SFA to the PT trunk using an autologous vein graft. Simultaneously, all toe amputation was performed, followed by a later metatarsal-level stump revision. Postoperative blood flow to the foot improved. Postoperative angiography confirmed good blood flow from the bypass graft through the tibial collaterals toward the foot. Although wound healing required time, the foot lesion was nearly healed by 3 months post-surgery.

#### CONCLUSION

We report on this case, including a review of the relevant literature.

### P1-4 Prophylactic Antibiotics in Clean Vascular Surgery: Reassessing the Necessity

Myeonghyeon Ko^1^ and Song-Yi Kim^1,2^

^1^Division of Vascular and Transplant Surgery, Department of Surgery, Chungnam National University Sejong Hospital, Korea

^2^Division of Vascular and Transplant Surgery, Department of Surgery, Chungnam National University College of Medicine, Korea

#### INTRODUCTION

The use of prophylactic antibiotics is included as an indicator in hospital quality evaluations. However, supporting evidence for this practice in clean vascular surgeries remains limited. This study aimed to evaluate whether avoiding routine prophylactic antibiotics increases the risk of surgical site infection (SSI) in such surgeries.

#### METHODS

We retrospectively reviewed 317 patients who underwent either native vascular access creation (arteriovenous fistula [AVF]) or varicose vein surgery at a single center between August 2020 and December 2025. Patients were categorized based on whether they received prophylactic antibiotics (n = 47) or not (n = 270). Baseline demographics and perioperative characteristics were compared. The primary outcome was the incidence of SSI, defined by the administration of postoperative antibiotics within 30 days.

#### RESULT

Only 1 SSI (0.3%) occurred in the entire cohort, in a patient who had not received prophylactic antibiotics. The antibiotic group was older (p = 0.011), had higher body mass index (p = 0.046), more diabetes (p = 0.040), and chronic kidney disease (p <0.001), with more AVF surgeries. Despite these differences, no increased SSI risk was observed in the group that omitted prophylactic antibiotics.

#### CONCLUSION

In our cohort, avoiding prophylactic antibiotics in clean vascular surgeries was not associated with increased SSI risk. These results suggest that routine prophylactic antibiotic use may have limited value in clean surgeries and support a more selective approach.

### P1-5 Surgical Reconstruction for Two Hepatic Artery Aneurysms with High Anatomical Complexity

Izumi Fukii, Shinsuke Kikuchi, Kyokei Fuchizawa, Hirofumi Jinno, Takayuki Uramoto, Seima Ohira, Keisuke Kamada, Naoya Kuriyama, Nobuyoshi Azuma

Department of Vascular Surgery, Asahikawa Medical University, Japan

#### INTRODUCTION

Although endovascular therapy (EVT) is considered the first treatment option for visceral artery aneurysms, open surgical reconstruction is recommended for anatomically complex cases.

#### METHODS

Case 1 involved a woman in her 70s with multiple proper hepatic artery aneurysms: a 28-mm fusiform aneurysm immediately distal to the gastroduodenal artery (GDA) origin and an additional 8-mm saccular aneurysm proximal to the bifurcation into the right and left hepatic arteries. EVT was deemed unsuitable because of a large inflow orifice and a short proximal neck. After aneurysmectomy, the arterial opening was closed by primary closure. The GDA was dissected distally, divided, and arterial reconstruction was achieved via end-to-end anastomosis between the GDA and the distal proper hepatic artery beyond the second saccular aneurysm. Case 2 involved a man in his 70s with a right hepatic artery aneurysm (maximum diameter 22 mm, saccular) arising immediately distal to the bifurcation into the GDA and left hepatic artery, with no proximal neck. Open surgery was selected to avoid embolization of these branches. Following aneurysmectomy, the divided right hepatic artery was reimplanted into the aneurysmectomy defect, using the resection opening itself as the anastomotic site.

#### RESULT

Postoperative courses were uneventful in both cases.

#### CONCLUSION

We report 2 cases of anatomically complex hepatic artery aneurysms successfully treated with aneurysmectomy and arterial reconstruction with favorable outcomes.

#### KEYWORDS

aneurysm, hepatic artery, vascular surgical procedure, revascularization, treatment outcome

### P1-6 Updated Nationwide Trends and Treatment Patterns of Abdominal Aortic Aneurysm in Korea (2018–2023)

Donghyun Lim, Jayeon Ahn, Ara Cho, Sangil Min, Seung-Kee Min, Jongwon Ha, Sanghyun Ahn

Division of Vascular Surgery, Seoul National University Hospital, Korea

#### INTRODUCTION

The epidemiology and management of abdominal aortic aneurysm (AAA) vary by region and ethnicity. Updated nationwide data on AAA trends in Korea are lacking since the previous report covering 2012–2016. This study aimed to analyze the current epidemiological trends, treatment patterns, and outcomes of AAA in Korea.

#### METHODS

A retrospective study was conducted using the Korean National Health Insurance Service database. All patients diagnosed with AAA from 2018 to 2023 were included and classified as intact AAA (iAAA) or ruptured AAA (rAAA). Treatment modalities included endovascular aneurysm repair (EVAR), open surgical repair (OSR), and conservative management. Primary outcomes were annual prevalence trends and 30-day mortality.

#### RESULT

A total of 33796 patients were analyzed (iAAA: 32404; rAAA: 1392; 78.5% male). Annual iAAA cases increased from 4373 (2018) to 4870 (2023), while rAAA remained stable; rAAA proportion decreased from 6.9% to 5.4%. Surgery was performed in 31.4% of iAAA (EVAR 68.7%, OSR 31.3%) and 59.9% of rAAA (EVAR 37.6%, OSR 61.2%). For iAAA, 30-day mortality was 1.8% (EVAR) and 4.0% (OSR). For rAAA, it was 35.0% (EVAR), 23.2% (OSR), and 89.1% (no surgery). High-volume centers showed lower rAAA-OSR mortality (17.3% vs. 28.1%).

#### CONCLUSION

AAA prevalence in Korea is increasing, while rAAA proportion has decreased. EVAR predominates for iAAA; OSR remains common for rAAA. Regional variations and volume-outcome relationships highlight the need for quality standardization.

### P1-7 Edema and Cardiac Arrhythmias in Spaceflight

Mitsumi Yamashita

Keihakukai Medical Corporation, Kannai Medical Clinic, Japan

#### INTRODUCTION

In recent years, interest in the effects of the space environment on the human body has grown in anticipation of long-term stays on the International Space Station (ISS) and future exploration of Mars. The cardiovascular system is particularly susceptible to the effects of microgravity and radiation, and arrhythmias and edema associated with fluid imbalances are considered important medical issues.

#### METHODS

This narrative review summarizes previous reports on the effects of the space environment on the development of arrhythmias and edema based on previously published studies.

#### RESULT

In microgravity, body fluids shift from the lower extremities to the head and thorax, causing changes in circulatory dynamics and autonomic nervous balance. Furthermore, facial edema is frequently observed early in spaceflight and is thought to be related to changes in venous return and lymphatic circulation. During long-term space missions, circulatory plasma volume decreases and changes in vascular regulation, and peripheral edema may appear after return.

#### CONCLUSION

Circulatory adaptation to the space environment may be involved in the development of arrhythmias and edema, and is an important issue in medical management during long-term space missions. These findings are expected to contribute not only to space medicine but also to our understanding of circulatory pathologies on Earth.

### P1-8 Long-Term Kissing Stent Outcomes for AIOD: Impact of Stent Type and Geometry

Jinsol Jung, Hyung Sub Park, Taeseung Lee

Seoul National University Bundang Hospital, Korea

#### INTRODUCTION

Reports on kissing stents (KS) with 10-year follow-up are limited. We analyzed 20-year outcomes and anatomical factors affecting long-term patency after KS placement for aortoiliac occlusive disease (AIOD).

#### METHODS

We retrospectively reviewed 53 patients (2004–2024). Variables included TASC II class, stent type (balloon-expandable [BE] vs. self-expanding [SE], diameter discrepancy, and proximal aortic protrusion length. Patency was estimated using Kaplan–Meier analysis.

#### RESULT

Maximum follow-up was 183 months. Mean ankle-brachial index improved from 0.51 ± 0.17 preoperatively to 0.97 ± 0.11 postoperatively (p <0.05). Primary patency at 1, 5, and 10 years was 89.5%, 72.4%, and 68.4%, respectively. Secondary patency remained high at 96.8%, 89.5%, and 89.5% at 1, 5, and 10 years. Patency loss occurred in 3 cases (6.4%), and reintervention was performed in 10 cases (21.3%). Procedure-related complications included puncture-site hematoma (n = 2), pseudoaneurysm (n = 2), and ventricular tachycardia (n = 1). Patency loss did not differ significantly between BE and SE stents (3.2% vs. 13.3%, p = 0.244) and was not associated with diameter discrepancy. Notably, all patency loss events occurred in patients with aortic protrusion >20 mm (13.6% vs. 0%, p = 0.078), suggesting a potential impact of excessive protrusion on long-term durability.

#### CONCLUSION

KS placement provides durable long-term results for AIOD. Proximal aortic protrusion length may be an important technical factor associated with long-term patency.

#### KEYWORDS

aortoiliac occlusive disease, kissing stent, stent protrusion

### P1-9 The Outcome of “Unplanned” Renal Vein Ligation During Abdominal Aortic Surgeries

Youseok Jeong Junyong Jekal Sangsu Lee

Pusan National University Yangsan Hospital, Korea

#### INTRODUCTION

Renal vein ligation can be performed during abdominal aortic surgeries to access the proximal anastomosis site and secure the margins for aortic clamping. Vessel ligation is usually decided during surgery, and it may affect renal function by restricting venous flow, but the results have rarely been investigated. This study aims to confirm the effect of renal vein ligation during the abdominal aortic surgery.

#### METHODS

This study used data from patients who previously underwent left renal vein ligation during open surgery of the abdominal aorta at Pusan National University Yangsan Hospital from 2021 to 2025. Among the patients who underwent renal vein ligation, the rates of occurrence of acute renal failure, chronic renal failure, and hemodialysis were investigated.

#### RESULT

A total of 179 open aortic surgeries were performed from 2021 to 2025 (142 abdominal aortic aneurysms, 37 Leriches syndrome). Seventeen patients underwent renal vein ligation during the surgery. Five patients experienced postoperative acute renal failure, but 3 of them recovered with fluid management. Two patients progressed to chronic renal failure and were treated with hemodialysis, but 1 of these patients had stage IV chronic renal failure, so the connection between the vein ligation and renal failure is unclear.

#### CONCLUSION

Although renal vein ligation may lead to renal failure, the procedure is assumed to be valid during aortic surgery even when not planned previously.

### P1-10 Age Thresholds in the Selection of EVAR versus OSR for Short Neck AAA: A Systematic Review

Young Jun Park

The Catholic University of Korea, Yeouido ST. Mary’s Hospital, Korea

#### INTRODUCTION

The optimal age threshold for selecting endovascular aneurysm repair (EVAR) versus open surgical repair (OSR) in short-neck infrarenal abdominal aortic aneurysms (AAA) remains unclear, particularly given age-related increases in perioperative risk.

#### METHODS

A systematic literature review was conducted according to PRISMA (Preferred Reporting Items for Systematic Reviews and Meta-Analyses) 2020 guidelines. Studies published between 2014 and 2025 comparing EVAR and OSR with age-stratified mortality outcomes were identified from major databases. Ten studies, including systematic reviews, observational cohorts, and 1 national registry, met the inclusion criteria. Mortality outcomes were assessed across perioperative and long-term follow-up.

#### RESULT

In octogenarians (≥80 years), EVAR was consistently associated with markedly lower perioperative mortality compared with OSR (pooled OR 0.23–0.28), while long-term survival was comparable between modalities. Registry data in nonagenarians (≥90 years) demonstrated prohibitive mortality with OSR and substantially lower early mortality with EVAR. In patients younger than 80 years, EVAR reduced perioperative mortality, but OSR showed comparable or superior long-term durability in selected low-risk individuals.

#### CONCLUSION

Age ≥80 years represents an evidence-based threshold favoring EVAR over OSR for short-neck infrarenal AAA. In patients <80 years, treatment should be individualized based on surgical risk, life expectancy, and anatomy.

## Poster Presentation II

**Moderators:**

Sang Dong Kim

Division of Vascular and Transplant Surgery, Department of Surgery, Incheon St. Mary's Hospital, The Catholic University of Korea, Incheon, Korea

Makoto Mo

Namiki Clinic, Japan

### P2-1 The Efficacy of Combined Treatment for Both Varicose Vein and Pelvic Varices

Yongbeom Bak

Cheongmac Vascular and Endovascular Hospital, Korea

#### INTRODUCTION

Many patients with venous disease experience persistent symptoms despite standard lower-limb treatment. The 2021 SVP (Symptoms-Varices-Pathophysiology) classification has expanded the diagnostic scope to include pelvic venous disorders. This study evaluates the efficacy of coil embolization for pelvic varices in relation to lower-limb therapies.

#### METHODS

Between September 2023 and December 2025, 815 patients underwent 1044 coil embolization procedures. Patients were grouped into concurrent treatment (n = 428), sequential treatment after prior surgery (n = 296), and isolated pelvic treatment (n = 91). Diagnosis utilized duplex ultrasonography and computed tomography, with at least 1 month of follow-up.

#### RESULT

All groups showed statistically significant symptomatic improvement. Notably, the sequential group—who had unresolved symptoms after previous varicose vein surgery—reported significant relief in lower-limb discomfort following pelvic embolization.

#### CONCLUSION

Pelvic varices and lower-limb varicose veins constitute a continuous disease spectrum. Following the 2022 ESVS (European Society for Vascular Surgery) guidelines, clinicians should integrate SVP and CEAP (Clinical-Etiology-Anatomy-Pathophysiology) classifications to ensure that pelvic origins of venous disease are addressed through meticulous examination and advanced imaging.

### P2-2 Successful Bypass Surgery for Left External Iliac Artery Occlusion Caused by Endofibrosis

Shuji Kurata

Shonan Fujisawa Tokushukai Hospital, Japan

#### INTRODUCTION

External iliac artery endofibrosis (EIAE) is a rare, non-atherosclerotic arteriopathy that mainly affects young endurance athletes. Repetitive hip flexion and mechanical stress during prolonged exercise are thought to cause intimal fibrosis and luminal narrowing, resulting in exertional claudication. Owing to its rarity, the optimal revascularization strategy remains unclear. We report a case of complete external iliac artery occlusion due to EIAE successfully treated with prosthetic bypass surgery.

#### METHODS

A 41-year-old man, a former Japanese national duathlon athlete, presented with progressive left intermittent claudication lasting 13 years. Computed tomography revealed complete occlusion of the left external iliac artery, and other causes such as atherosclerosis and thrombotic disease were excluded. EIAE was diagnosed. Considering the complete occlusion and the need for long-term durability, a left common iliac artery–common femoral artery bypass using a prosthetic graft was performed.

#### RESULT

The postoperative course was uneventful. Symptoms resolved completely, limb perfusion improved, and the patient returned to full physical activity.

#### CONCLUSION

EIAE should be considered in young athletes with exertional leg symptoms. Prosthetic bypass surgery may be a durable treatment option for advanced EIAE. This is the first reported bypass case for EIAE in Japan.

**Figure:**
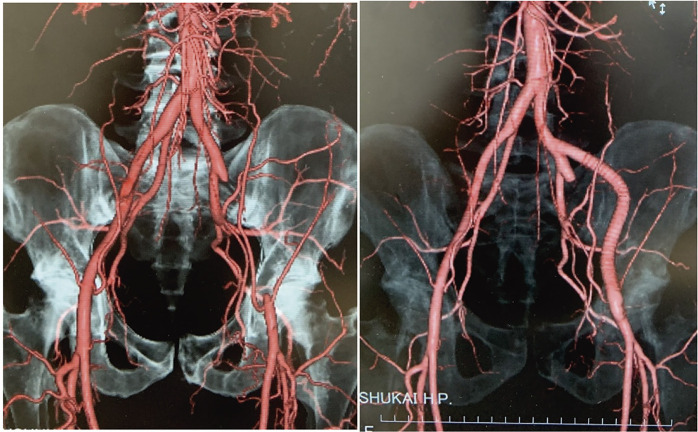


### P2-3 Impact of Metabolic Activity on Abdominal Aortic Aneurysm Growth Evaluated by ^18^F-FDG PET–CT

Hye-Won Jang, MD, Sang Ah Lee, MD, Jun Gyo Gwon, MD, PhD, Youngjin Han, MD, PhD, Yong-Pil Cho, MD, PhD

Department of Vascular Surgery, University of Ulsan College of Medicine, Asan Medical Center, Seoul, Republic of Korea

#### INTRODUCTION

Aortic wall inflammation has been suggested as a factor influencing aneurysm progression; however, proving its relationship with actual aneurysmal growth rate is limited. This study aimed to evaluate the metabolic activity of abdominal aortic aneurysms (AAAs) using fluorine-18 fluorodeoxyglucose positron emission tomography–computed tomography (^18^F-FDG PET–CT) and to investigate its association with aneurysm growth.

#### METHODS

This retrospective, single-center study included 182 patients who underwent ^18^F-FDG PET–CT between January 2014 and December 2024 and were diagnosed with untreated infrarenal fusiform AAAs. Patients were classified into growth (n = 98) and nongrowth (n = 84) groups according to baseline aneurysm size and initial 1-year growth rate. maximum mean standardized uptake value AAA (SUVmean_AAA) and maximum standardized uptake value AAA (SUVmax_AAA) were measured at the aneurysmal wall, and the SUV ratio was defined as SUVmean_AAA divided by the SUVmax of the descending thoracic aorta.

#### RESULT

The growth group demonstrated a significantly higher SUV ratio compared with the nongrowth group (1.01 ± 0.15 vs. 0.95 ± 0.17, p = 0.01). In contrast, SUVmean_AAA (2.59 ± 0.46 vs. 2.55 ± 0.41, p = 0.57) and SUVmax_AAA (2.63 ± 0.57 vs. 2.56 ± 0.56, p = 0.44) showed no significant differences. The SUV ratio was positively correlated with the 1-year aneurysm growth rate (r = 0.26, p <0.001) and the 5-year mean growth rate (r = 0.19, p = 0.01).

#### CONCLUSION

The SUV ratio measured by ^18^F-FDG PET–CT showed a significant association with AAA growth. These findings suggest that assessing AAA metabolic activity may help predict subsequent growth rate and determine therapeutic strategies.

**Figure:**
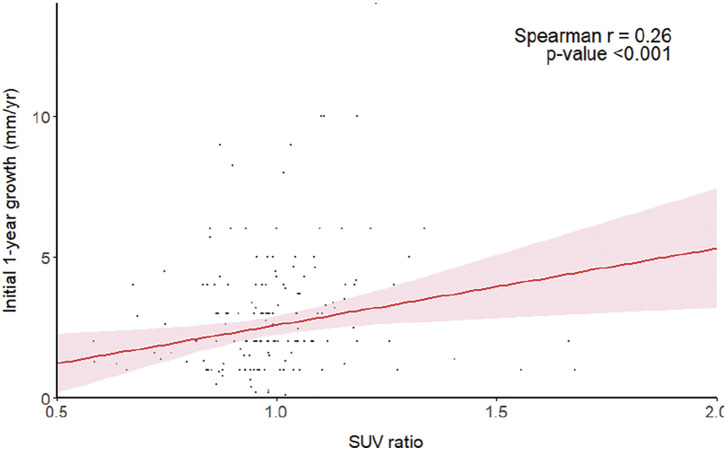


**Figure:**
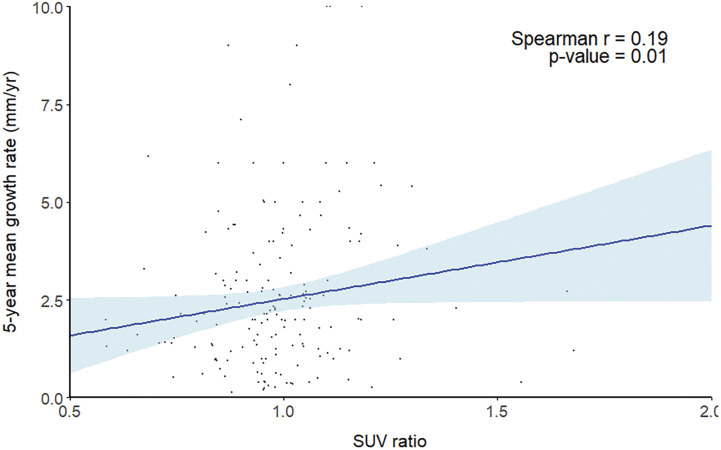


### P2-4 Successful Treatment of Endoleak-Induced DIC after TEVAR: A Case Report

Hiraku Kamimura, Takuro Shirasu, Takumi Miwa, Takayuki Sawai, Tomomi Nawa, Kai Suzuki, Kozue Watabe, Natsumi Iijima, Daisuke Mochizuki, Yu Tadokoro, Kenshiro Kawabe, Ayako Takasaka, Masaya Sano, Tosh

Division of Vascular Surgery, Department of Surgery, The University of Tokyo, Japan

#### INTRODUCTION

Disseminated intravascular coagulation (DIC) can occur in patients with aortic aneurysms or after endovascular aneurysm repair complicated by endoleak. Once established, DIC may be life-threatening, and definitive treatment requires correction of the underlying aneurysm pathology or endoleak. We report a case of DIC triggered by an endoleak after thoracic endovascular aortic repair (TEVAR) that was successfully treated with redo TEVAR.

#### METHODS

An 80-year-old man with a descending aortic aneurysm was treated with TEVAR 3 years previously. Follow-up computed tomography (CT) showed a type 1b endoleak, and a percutaneous redo TEVAR was performed. However, CT on postoperative day 4 (POD 4) revealed a new type 1a endoleak. In addition, late-onset pseudoaneurysm at the puncture site was suspected. Therefore, further redo TEVAR and pseudoaneurysm repair were planned. Thrombocytopenia developed 3 days before the scheduled surgery and progressively worsened until the day of operation. Based on the clinical course and laboratory findings, DIC induced by the persistent endoleak was suspected.

#### RESULT

Redo TEVAR was carried out as planned. Postoperatively, factor XIII supplementation and administration of tranexamic acid were performed. Following exclusion of the endoleak, platelet counts and coagulation parameters improved, and the patient was discharged on POD 7 uneventfully.

#### CONCLUSION

In this case, DIC induced by a type 1a endoleak was successfully resolved by redo TEVAR. Prompt correction of an endoleak is crucial when DIC develops after endovascular aortic repair.

#### KEYWORDS

DIC, TEVAR, endoleak

**Figure:**
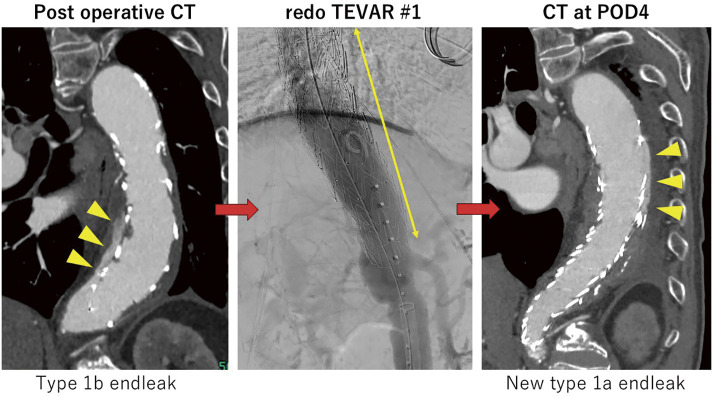


**Figure:**
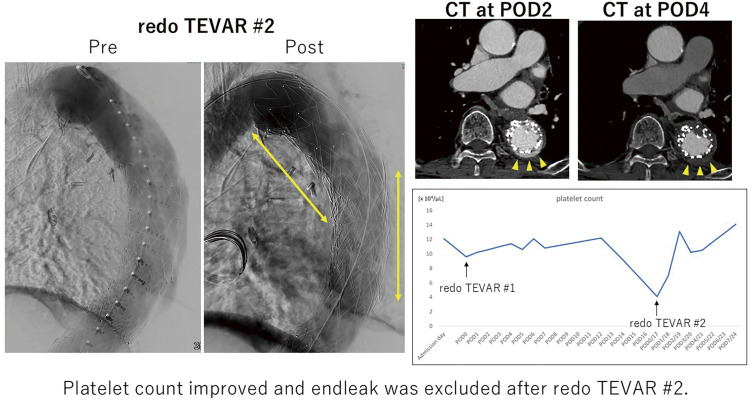


### P2-5 Long-Term Outcomes after Use of Cyanoacrylate Closure to the Venous Leg Ulcer

Hyangkyoung Kim^1^, Jin Hyun Joh^2^, Sungsin Cho^2^

^1^Department of Surgery, Ewha Womans University Mokdong Hospital, Seoul, Korea

^2^Department of Surgery, Kyung Hee University Hospital at Gangdong, Seoul, Korea

#### INTRODUCTION

Venous leg ulcers (VLUs) represent a chronic and costly global health challenge, significantly impacting patient quality of life due to high recurrence rates often stemming from underlying venous incompetence. Effective treatment strategies targeting venous reflux are crucial for successful VLU management.

#### METHODS

This study analyzed a prospectively collected database (2017–2024) of 822 cyanoacrylate glue closure cases for venous disease. A 1:5 propensity score matching process was applied to balance baseline demographics between 23 ulcer-positive patients and a matched cohort of 115 ulcer-negative patients, facilitating a robust comparison. We assessed baseline characteristics, clinical and anatomical findings, and recurrence-free survival using Kaplan–Meier analysis, with p-values reported for group comparisons.

#### RESULT

The ulcer-positive patients exhibited significantly higher rates of night cramping (34.8% vs. 13.9%, p = 0.016), deep vein reflux (52.2% vs. 12.2%, p <0.001), and perforator reflux (39.1% vs. 4.3%, p <0.001). Overall recurrence-free survival was 70.4% at 24 months, decreasing to 48.3% after 36 months. While patients without venous reflux showed visually better recurrence-free survival, these differences were not statistically significant in stratified analyses.

#### CONCLUSION

Cyanoacrylate closure is highlighted as an effective method for treating VLUs caused by venous incompetence. While the study notes that stratified recurrence-free survival rates did not reach statistical significance, the clinical outcomes remain highly promising.

### P2-6 A Case of a Perigraft Seroma after Axillo-Bifemoral Bypass Using ePTFE Prosthetic Vascular Graft

Yukina Hayashi, Toshio Takayama, Ayako Takasaka, Shinogu Hatano, Taku Kamimura, Wataru Sakai, Masaya Sano, Takuro Shirasu, Katsuyuki Hoshina

Department of Vascular Surgery, The University of Tokyo Hospital, Japan

#### BACKGROUND

Seroma formation around prosthetic vascular grafts is a known complication; however, its pathogenesis and optimal management strategy remain unclear.

#### CASE

**Fig. 1:**
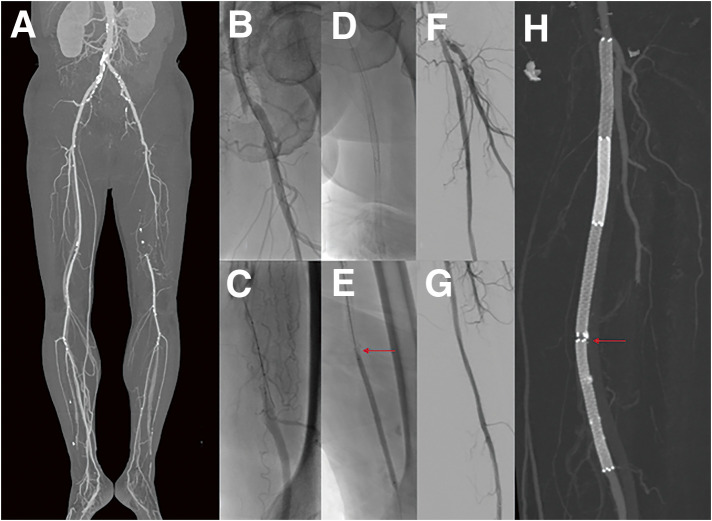
(**A**) Preoperative CT angiogram revealing a long SFA occlusion measuring 25 cm, with reconstitution distally at the proximal popliteal artery. (**B**) From a right-side up-and-over approach, the angiogram revealed proximal SFA occlusion with a visible orifice of SFA. (**C**) The antegrade approach failed to re-enter distally, necessitating retrograde puncture. (**D**) Proximal SFA: Eluvia stent (7 × 120 mm); mid-SFA: Eluvia stent (6 × 120 mm). (**E**) Short overlap (red arrow) between the mid-SFA stent and distal SFA stent (6 × 80 mm). (**F**–**G**) Final angiogram revealing good stent expansion and hemostasis of the retrograde puncture in the P1 segment. (**H**) Follow-up CTA at day 4 revealing stent migration causing a stent-free lesion (red arrow) between the distal 2 stents.

A 72-year-old man underwent open repair for an abdominal aortic aneurysm and left internal iliac artery aneurysm at the age of 64. Four years later, he developed abdominal aortic occlusion caused by acute Stanford type B aortic dissection and underwent an axillo-bifemoral bypass using an expanded polytetrafluoroethylene (ePTFE) graft (Fig. 1A).

#### RESULT

**Fig. 2:**
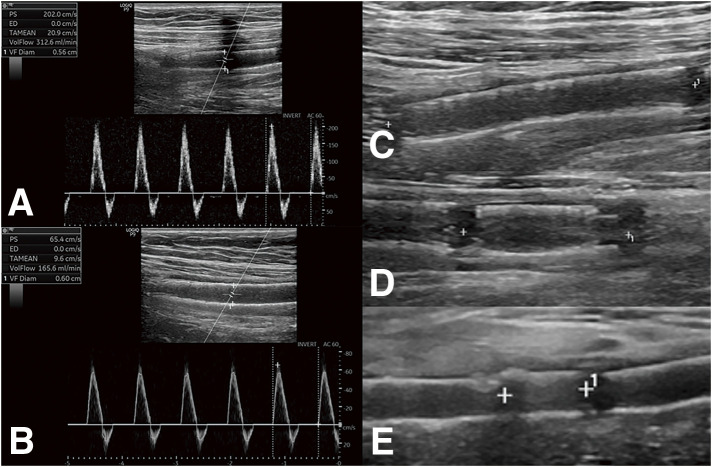
(**A**) DUS on outpatient follow-up at 5 months revealed elevated PSV of 202 cm/s at the stent-migration lesion. **B**) The normal segment at the mid-SFA stent shows a PSV of 65.4 cm/s, resulting in a PSV ratio of 3.1. (**C**–**E**) Multiple stent fractures were noted on DUS at the mid- to distal SFA stents.

A seroma around the prosthetic graft was identified in the right groin early after the surgery. As there were no signs of infection, conservative management was selected; however, the seroma gradually increased in size over time. When the maximum diameter reached 117 mm (Fig. 1B), severe skin tension became apparent (Fig. 2A). Also, duplex ultrasound demonstrated an increased peak systolic velocity at the graft-to-graft anastomosis. Surgical intervention was planned to prevent skin breakdown and possible graft infection. The seroma contents were removed (Fig. 2B), and a poorly organized segment of the graft was partially resected (Fig. 2C) and replaced with an 8-mm ePTFE graft (Fig. 2D).

#### CONCLUSION

Surgical removal of the seroma contents and partial graft replacement were effective for treating a giant perigraft seroma and resulted in a favorable outcome.

**Figure:**
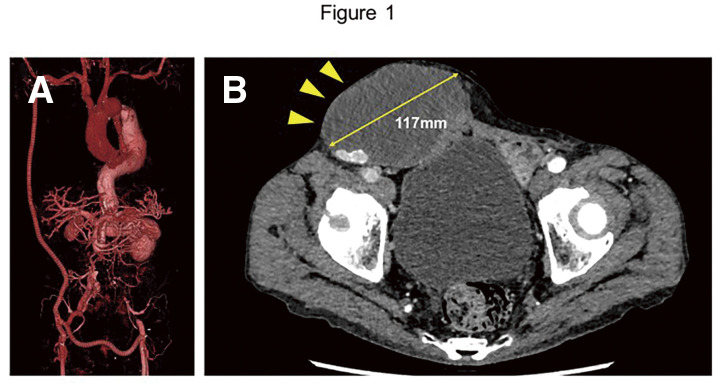


**Figure:**
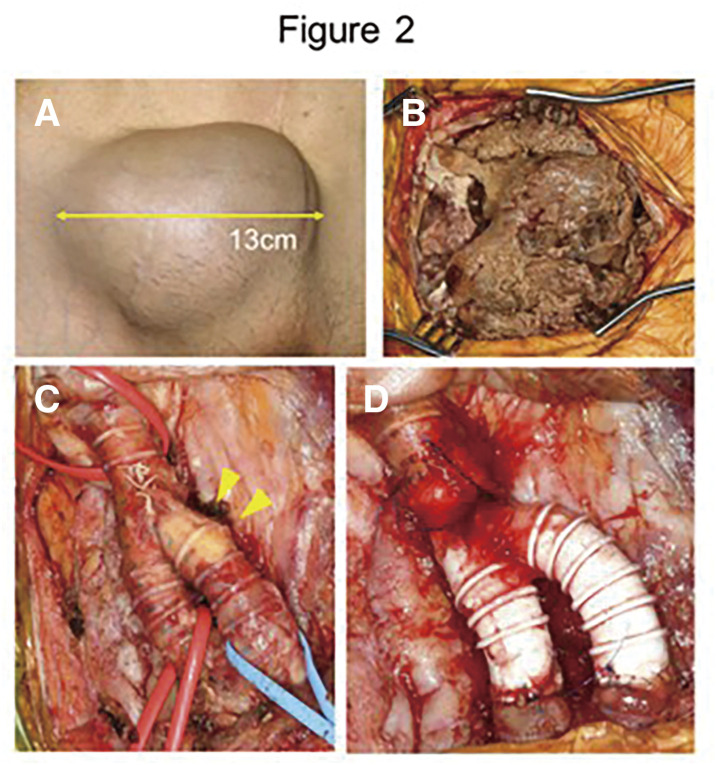


### P2-7 Clinical Feasibility of Chimney EVAR as a Salvage for Failed EVAR and Ruptured AAA

HaengJin OHE, Ki Yoon Moon, Eun Ju Jang, Jang Yong Kim, Sun Cheol Park, Sang Seob Yun

Seoul St. Mary's Hospital, Korea

#### INTRODUCTION

This study evaluates the technical feasibility and clinical outcomes of the chimney endovascular aneurysm repair (Ch-EVAR) technique as a salvage strategy for failed EVAR and emergent aortic rupture.

#### METHODS

We retrospectively reviewed 5 patients (mean age: 82 years) treated with Ch-EVAR between 2018 and 2025. Indications included ruptured abdominal aortic aneurysm (n = 3) and sac expansion due to type 1a endoleaks from prior EVAR (n = 2). Targeted branches for preservation were the right renal artery (n = 3), left renal artery (n = 2), and superior mesenteric artery (n = 1). Stents (Lifestream, Begraft, Express LD) were utilized as chimney grafts. Aortic stent grafts were deployed with a mean oversizing of 23.5% (range: 7.7%–33.3%).

#### RESULT

Technical success was achieved in 100% of cases (5/5). The mean procedural time was 149.6 min. While 2 patients exhibited immediate post-procedural gutter leaks, these resolved spontaneously within 7 days. Over a mean follow-up of 338 days, sac diameters remained stable in most patients. One patient required re-intervention for gutter embolization.

#### CONCLUSION

Ch-EVAR is a feasible “off-the-shelf” salvage modality for complex aortic pathologies and failed EVAR. Meticulous sizing and oversizing are essential to mitigate gutter leaks and ensure technical success in high-risk patients.

### P2-8 Aneurysm Reduction and Autogenous Patch Angioplasty in a Patient with Hemodialysis Access Aneurysm

Soon Cheon Lee

Division of Vascular Surgery, Gwang yang Sarang Hospital, Korea

#### INTRODUCTION

This study aimed to evaluate the effectiveness of partial aneurysm resection and reduction combined with autologous vein patch angioplasty for concomitant outflow vein stenosis in hemodialysis vascular access–related aneurysms. In addition, we investigated the therapeutic impact of concomitant inflow flow reduction procedures on inflow–outflow mismatch.

#### METHODS

We retrospectively reviewed 33 cases of hemodialysis access–related aneurysms treated between January 2021 and December 2025 by a single surgeon. All patients underwent partial aneurysm resection and reduction with autologous vein patch angioplasty for associated outflow vein stenosis. Evaluated parameters included the anatomical locations of aneurysms and outflow stenosis, the flow reduction effect of inflow flow reduction procedures, restenosis at the patch angioplasty segment, frequency of postoperative reinterventions, occurrence of new stenotic lesions, mean patch length, surgical complications, the most recent access flow volume, and the development of new aneurysms

#### RESULT

The mean follow-up duration was 26.6 months (range, 4–52 months). All 33 patients underwent partial aneurysm resection and reduction with autologous vein patch angioplasty. One patient experienced delayed wound healing at the aneurysm resection site, requiring resuturing. A total of 33 reinterventions were performed in 13 patients. Stenosis at the patch angioplasty site occurred in 4 patients, while 9 patients developed new stenotic lesions; some patients required multiple reinterventions. During follow-up, 2 patients died; however, no vascular access abandonment occurred, and no new aneurysms developed. Inflow flow reduction procedures were performed in 11 patients, resulting in a decrease in mean access flow from 1770 mL/min before reduction to 862.5 mL/min after reduction. Access configurations included radiocephalic arteriovenous fistula (AVF) in 20 cases, brachiocephalic AVF in 10 cases, brachiobasilic AVF in 2 cases, and ulnobasilic AVF in 1 case. The length of the autologous vein patch ranged from 2 to 6 cm, with a width of 1.5 to 2 cm. Excluding 4 patients without flow measurements, the mean latest measured access flow volume in 29 patients was 891 mL/min.

#### CONCLUSION

Partial aneurysm resection and reduction combined with autologous vein patch angioplasty for outflow vein stenosis is a durable and predictable treatment strategy for hemodialysis access–related aneurysms. When combined with inflow flow reduction procedures, this approach effectively improves inflow–outflow mismatch.

#### KEYWORDS

hemodialysis access aneurysm reduction, autogenous patch angioplasty, inflow outflow mismatch

### P2-9 Comparative Stent Patency after Iliac Vein Stenting for Iliac Vein Occlusion of Different Etiologies

Suehyun Park, Hyung-Kee Kim, Seung Huh, and Deokbi Hwang

Kyungpook National University Hospital, Korea

#### INTRODUCTION

Iliac vein obstruction is commonly treated with stenting; however, differences in underlying pathology, including acute and chronic deep vein thrombosis (DVT) and non-thrombotic iliac vein lesions (NIVL), may affect stent patency. This study compared stent patency according to etiology.

#### METHODS

This retrospective, 2-center study included 79 patients who underwent iliac vein stent insertion between 2011 and 2025. Four patients were lost to follow-up and excluded, leaving 75 patients for analysis (62 acute DVT, 11 chronic DVT, and 2 NIVL). Primary patency was defined as freedom from in-stent restenosis or occlusion, and secondary patency as freedom from stent occlusion. Patency was assessed using follow-up imaging and clinical evaluation.

#### RESULT

The 1- and 2-year primary patency rates were 82.7% and 80.3% in the acute DVT group and 63.6% and 63.6% in the chronic DVT group, respectively. Primary patency was maintained at 100% at 1 and 2 years in the NIVL group (p = 0.062). The 1- and 2-year secondary patency rates were 88.2% and 85.6% in the acute DVT group and 72.7% and 72.7% in the chronic DVT group, while secondary patency was preserved in all NIVL patients (p = 0.307).

#### CONCLUSION

Although not statistically significant, iliac vein stenting in patients with chronic DVT showed a trend toward lower primary and secondary patency compared with acute DVT and NIVL. These findings suggest that chronic post-thrombotic changes may negatively affect stent durability and warrant careful surveillance after stent placement.

## E-Poster Exhibition

### EP-1 Risk Factors for Major Pulmonary Complications following Open AAA Surgery

Sang Ah Lee, MD, Jun Gyo Gwon, MD, PhD, Yong-Pil Cho, MD, PhD, Youngjin Han, MD, PhD

Division of Vascular Surgery, Department of Surgery, Asan Medical Center, University of Ulsan College of Medicine, Seoul, Korea

#### INTRODUCTION

Postoperative pulmonary complications remain a major cause of morbidity after open abdominal aortic aneurysm (AAA) repair. The independent predictive value of preoperative spirometry in this setting is unclear. We evaluated clinical, pulmonary, and surgical predictors of pulmonary complications after open AAA surgery.

#### METHODS

This single-center retrospective study included patients who underwent open AAA repair between 2011 and 2015. The primary outcome was postoperative pulmonary complications, defined as ventilator care ≥48 h or reintubation. Univariable and multivariable logistic regression analyses were performed with restricted covariate selection due to limited events.

#### RESULT

Among 187 patients, 20 (10.7%) developed pulmonary complications. On multivariable analysis, age (odds ratio [OR] 1.12 per year, 95% confidence interval [CI] 1.04–1.21, P = 0.002), body mass index (OR 1.23 per kg/m^2^, 95% CI 1.04–1.46, P = 0.015), and transfusion requirement (OR 1.28 per unit, 95% CI 1.10–1.50, P = 0.002) were independent predictors. Preoperative forced expiratory volume in 1 s <50% was not independently associated (OR 1.17, P = 0.897).

#### CONCLUSION

Pulmonary complications after open AAA repair were more strongly associated with surgical burden and patient factors than with spirometric severity.

#### KEYWORDS

abdominal aortic aneurysm, open surgical repair, postoperative pulmonary complications

**Figure:**
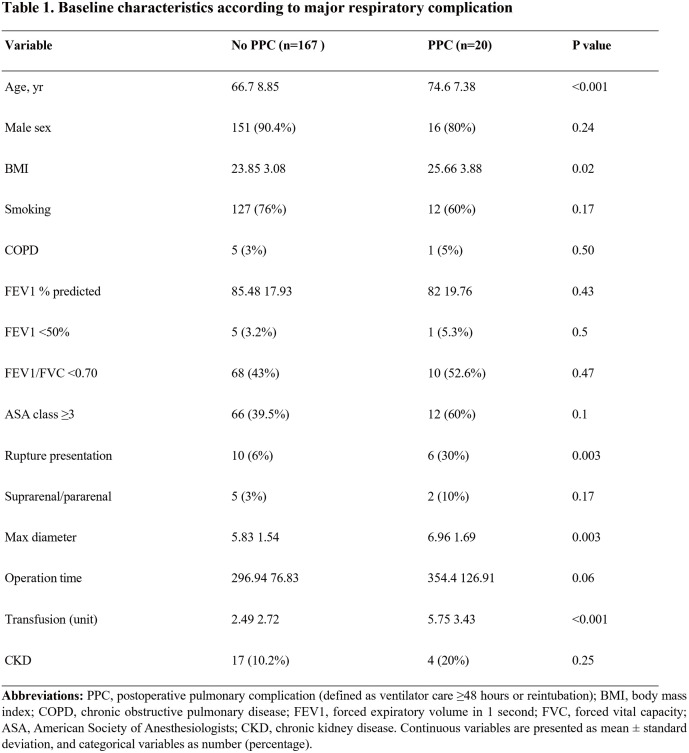


**Figure:**
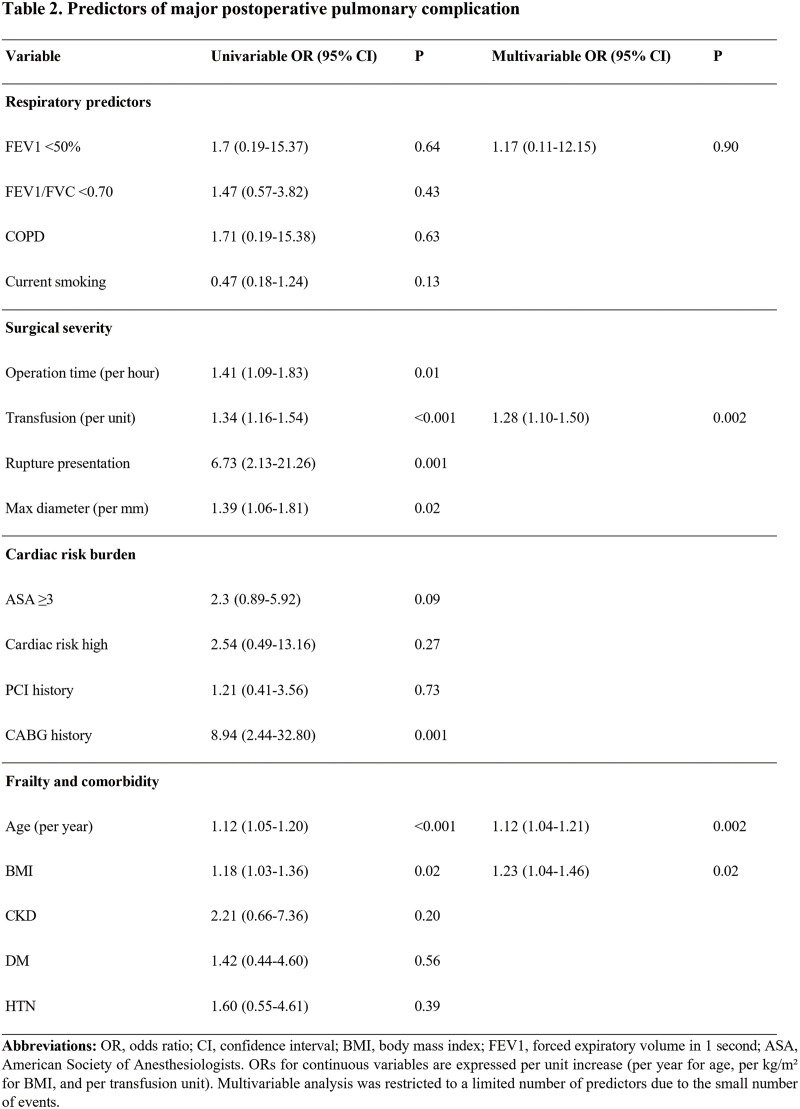


### EP-2 Early Fracture and Migration of DES Complicated by Recurrent Stent Fracture During Reintervention

Young Sup Yoo, MD

Department of Vascular Surgery, Design Hospital, Jeonju, Korea

#### INTRODUCTION

Endovascular treatment with drug-eluting stents (DES) is widely used for the treatment of superficial femoral artery (SFA) disease. Stent fracture in the SFA can occur within the first year of placement due to high mechanical stress, longer stent length, and overlapping stents. Early stent migration in the SFA is rare but significant and is often associated with improper vessel sizing, inadequate expansion, and excessive mechanical forces. Stent fractures and migration can lead to pseudoaneurysm formation, in-stent restenosis, or occlusion.

#### METHODS

A 59-year-old man presented with severe claudication in the left leg. He had a history of hypertension, diabetes mellitus, dyslipidemia, and cerebrovascular accident 1 year prior. He was initially treated with cilostazol and exercise therapy but failed to improve symptoms. Ankle-brachial index was 0.33 and computed tomography angiography (CTA) revealed long SFA occlusion. He underwent endovascular stenting with 3 Eluvia stents. CTA performed on day 4 after intervention revealed stent migration in the distal SFA. During follow-up, duplex ultrasound (DUS) was performed to monitor restenosis at the stent migration lesion. At 5 months, DUS revealed multiple stent fractures and an elevated peak systolic velocity ratio of 3.1 at the migration lesion; therefore, reintervention was planned.

#### RESULT

During reintervention, unexpected stent fracture of the proximal SFA stent occurred, resulting in migration of the fractured stent to the mid-SFA, which was treated with additional stenting.

#### CONCLUSION

Early fracture and migration can lead to restenosis and may be complicated by additional fracture during reintervention. Close surveillance and careful planning are crucial.

#### KEYWORDS

stent fracture, stent migration, stent embolization, drug-eluting stent, restenosis

**Fig. 3:**
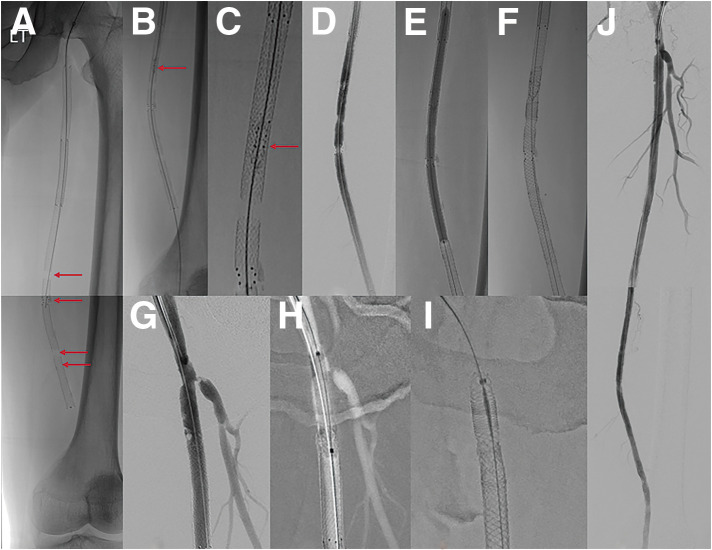
(**A**) Multiple stent fractures and stent migration lesions (red arrows) were noted at the beginning of reintervention. (**B**, **C**) The newly fractured and embolized stent segment (red arrow) from the proximal SFA stent to the midportion of the 2nd stent was found only after angiograms. (**D**) Angiogram revealing stenosis at the stent-fracture site, migration lesion, and embolized lesion. (**E**) After POBA with a 6-mm noncompliant balloon, DCB (ranger 6 × 150 mm) angioplasty was done covering all stent fracture sites, migration lesion, embolized lesion. (**F**) 6.5 × 100 mm Supera stenting was done covering all lesions. (**G**) About 2 cm length of stent missing at the proximal SFA with suspected dissection at the new stent edge. (**H**) After POBA with a 7-mm noncompliant balloon, DCB (INPACT 7 × 40 mm) angioplasty was done. (**I**) 7.5 × 60 mm Supera stenting was done. (**J**) Final angiogram revealed improved stenosis and well expansion of stents.

### EP-3 Surgical Treatment of Popliteal Artery Aneurysm Due to Type II Popliteal Artery Entrapment Syndrome

JaeKyung Shin, Jayeon Ahn, Donghyun Lim, Ara Cho, Sanghyun Ahn, Sangil Min, Seung-Kee Min

Division of Vascular Surgery, Seoul National University Hospital, Seoul, Korea

#### INTRODUCTION

Popliteal artery entrapment syndrome (PAES) is a rare vascular disorder caused by abnormal musculotendinous anatomy leading to chronic extrinsic compression of the popliteal artery. Persistent compression may result in progressive stenosis, thrombosis, distal embolization, or aneurysmal degeneration. We report a case of a symptomatic popliteal artery aneurysm (PAA) secondary to type II PAES.

#### METHODS

A 61-year-old man presenting with chronic right popliteal pain and a pulsatile mass was evaluated. Clinical findings, computed tomography angiography (CTA), and operative records were retrospectively reviewed. CTA was used to assess arterial pathology and its relationship to surrounding musculotendinous structures. Open surgical treatment was planned based on imaging findings.

#### RESULT

CTA revealed a right PAA and direct compression of the artery by an aberrant medial head of the gastrocnemius muscle, consistent with type II PAES. Open surgery was performed successfully, including myotendinous sectioning of the aberrant muscle, aneurysm resection, and interposition grafting with a reversed small saphenous vein. Adequate restoration of arterial flow was achieved, and no perioperative complications occurred.

#### CONCLUSION

PAA secondary to PAES is a rare but clinically significant condition. Definitive treatment requires correction of both the aneurysmal lesion and the causative musculotendinous abnormality. This case highlights the importance of considering PAES as an underlying etiology of PAA, even in older patients.

#### KEYWORDS

popliteal artery entrapment syndrome, popliteal artery aneurysm, gastrocnemius muscle, vascular reconstruction, reversed small saphenous vein interposition graft

**Figure:**
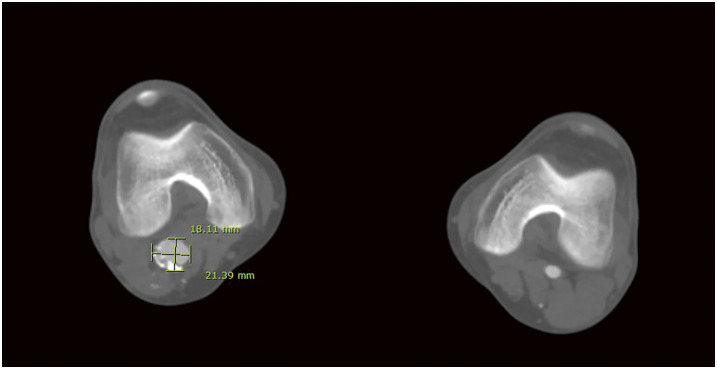


**Figure:**
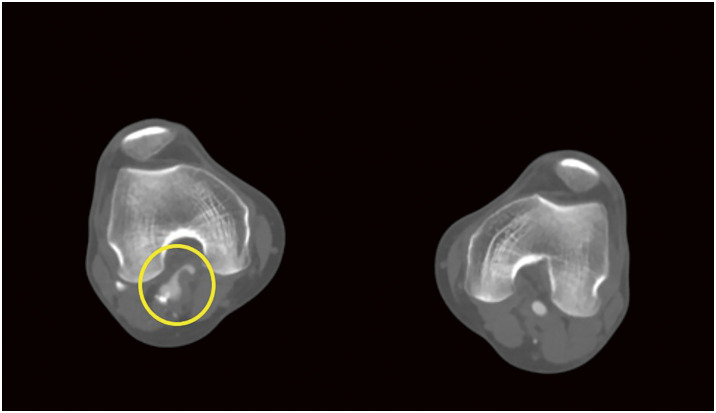


### EP-4 An Unusually Large Superficial Femoral Artery Aneurysm: A Case Report

Yohei Yamamoto, Kazuki Tsukuda, Natsuho Maekawa, Ai Kazama, Yoshiki Wada, Tsuyoshi Ichinose, Toshifumi Kudo

Division of Vascular Surgery, Department of Cardiovascular Surgery, Institute of Science Tokyo, Japan

#### INTRODUCTION

Aneurysms of the femoral arteries are uncommon, and isolated aneurysms of the superficial femoral artery (SFA) are even rarer. Herein, we report a case of an isolated large SFA aneurysm successfully treated with staged management.

#### METHODS

The patient was a 54-year-old man with a 2-year history of a mass in the right thigh. He presented with a recent rapid enlargement of the mass accompanied by pain.

#### RESULT

Computed tomography revealed a 16-cm aneurysm of the right SFA. It also demonstrated stenosis of the tibioperoneal trunk, which was suspected to be caused by distal embolization. Due to the risk of rupture and further distal embolization, urgent intervention was deemed necessary. Given the patient’s reduced ejection fraction and poorly controlled diabetes mellitus, endovascular treatment was selected as the initial therapeutic strategy. The patient underwent endovascular repair using 2 Viabahn stent grafts (8 × 250 mm and 9 × 100 mm). After adequate control of his comorbid conditions, a subsequent bypass surgery from the SFA to the posterior tibial artery was performed. The postoperative course was uneventful.

#### CONCLUSION

Aneurysms of the SFA are rare. To our knowledge, this case represents one of the largest SFA aneurysms reported in the literature. This case highlights that staged management incorporating endovascular treatment can be a feasible therapeutic option for selected patients with large SFA aneurysms.

#### KEYWORDS

superficial femoral artery, femoral artery aneurysm, endovascular procedures, bypass surgery, case reports

### EP-5 High Prevalence of IFU-Incompatible Anatomy in Ruptured Abdominal Aortic Aneurysm

Seong Jun Lim, MD, PhD, Su Ji Oh, MD, Hae Lee, MD, Myeong Su Kim, MD, Suk-Won Song, MD, PhD

Department of Thoracic and Cardiovascular Surgery, Ewha Womans University Medical Center, Ewha Womans University College of Medicine, Korea

#### INTRODUCTION

Endovascular repair for ruptured abdominal aortic aneurysm (rAAA) has been increasingly adopted; however, its applicability is limited by anatomical constraints defined by device instructions for use (IFU). We evaluated the prevalence of IFU-incompatible anatomy in patients undergoing open repair for rAAA at our institution.

#### METHODS

We retrospectively reviewed consecutive patients who underwent open surgical repair for rAAA. Preoperative computed tomography angiography was analyzed to assess anatomical suitability for endovascular repair. IFU incompatibility was defined as the presence of any of the following: proximal seal unsuitability (neck length ≤10 mm, diameter ≥32 mm, angulation ≥60°, or conical neck), distal seal unsuitability (common iliac artery landing length ≤15 mm or diameter ≥25 mm), or inadequate access (external iliac artery diameter ≤6 mm).

#### RESULT

Seventy patients were included in the analysis. Overall, 50 patients (71.4%) demonstrated IFU-incompatible anatomy for endovascular aneurysm repair (EVAR). Proximal sealing was unsuitable in 37 patients (52.9%), distal sealing in 25 (35.7%), and access was inadequate in 5 (7.1%). Only 15 patients (21.4%) met all anatomical criteria for EVAR feasibility.

#### CONCLUSION

A high prevalence of IFU-incompatible anatomy was observed among patients presenting with rAAA, most commonly related to unfavorable proximal neck morphology. These findings highlight the anatomical limitations that may restrict the applicability of standard EVAR in the emergent setting.

### EP-6 Vascular Health Status of Residents Aged ≥50 Years in Goseong-gun, Gangwondo

Su-kyung Kwon^1^ Jang Yong Kim^2^

^1^Seoul Medical Center, Seoul, Korea

^2^The Catholic University of Korea, Seoul St. Mary's Hospital, Seoul, Korea

#### INTRODUCTION

As the elderly population grows, the prevalence of chronic conditions such as hypertension and diabetes increases, raising the risk of cardiovascular and peripheral vascular diseases. This study evaluated vascular health in an aging region using a community-based screening program for early detection of asymptomatic disease.

#### METHODS

A cross-sectional study included 812 residents aged ≥50 years who participated in a vascular screening program in Goseong County between June 2022 and September 2025. Noninvasive assessments included carotid ultrasound, abdominal aortic ultrasound, and ankle–brachial index (ABI). Health surveys and vital measurements were collected. Descriptive analyses were performed to characterize vascular abnormalities in this single-region population.

#### RESULT

Among 812 participants (mean age 68.4 ± 8.2 years; 61.8% female), hypertension, diabetes, and dyslipidemia were prevalent at 65.9%, 26.3%, and 61.6%, respectively, with high rates of pharmacologic treatment. Despite this, noninvasive imaging revealed a significant burden of asymptomatic disease. Abnormal carotid ultrasound findings were detected in 49.9% (estimated >8000 residents). Abdominal aortic or iliac aneurysms were identified in 1.7%, and peripheral arterial disease (PAD) (ABI <0.9) in 1.0%.

#### CONCLUSION

A substantial burden of asymptomatic structural vascular disease was identified in this aging regional population. Noninvasive vascular screening may play an essential role in aging regions, where prevention should precede symptom-driven care.

#### KEYWORDS

vascular screening, carotid artery diseases, abdominal aortic aneurysm, peripheral arterial disease, aged

**Table 1:** Baseline characteristics and vascular screening results (n = 812)

Category	Variables	Value
Study population	Mean age (years)	68.4 ± 8.2
	Age distribution	50–59: 15.2% 60–69: 38.5% 70–: 46.3%
	Sex (male:female)	38.2%:61.8%
Comorbidities	Hypertension	65.9%
	Diabetes mellitus	26.3%
	Dyslipidemia	61.6%
	Current or former smoking	26.9%
Vital signs and measurements	Systolic blood pressure (mmHg)	132.5 ± 16.2
	Diastolic blood pressure (mmHg)	77.3 ± 10.0
	Blood glucose (mg/dL)	134.8 ± 44.4
	Body mass index (kg/m^2^)	24.7 ± 3.2
Vascular screening findings	Abnormal carotid ultrasound	49.9% (≈7766–8919 residents)
	Abdominal aortic or iliac aneurysm (AAA/IAA)	1.7% (≈176–483 residents)
	ABI-defined peripheral arterial disease	1.0% (≈86–332 residents)

### EP-7 Surgical Treatment of Nutcracker Syndrome Causing Severe Varicocele and Intermittent Hematuria

Hyojoon Kim, Jayeon Ahn, Donghyun Lim, Ara Cho, Sanghyun Ahn, Sangil Min and Seung-Kee Min

Division of Vascular Surgery, Seoul National University Hospital, Seoul, Korea

#### INTRODUCTION

Nutcracker syndrome (NCS) is a rare vascular disorder caused by outflow obstruction of the left renal vein (LRV) into the inferior vena cava. It causes LRV hypertension with subsequent renal pathology and eventual gonadal vein reflux with pelvic disorders in patients. We report a case of a 59-year-old man presenting with severe left scrotal varicocele and intermittent hematuria. Computed tomography (CT) showed nutcracker phenomenon with renal vein compression between the aorta and the superior mesenteric artery. The left gonadal vein was enlarged, causing varicosity in the left scrotum and left inguinal area. Coronal and sagittal CT images showed the compression and refluxed flow progression down to the scrotum and inguinal area.

#### RESULT

Open surgery was performed successfully, including LRV transposition to the distal vena cava, varicocelectomy, and great saphenous vein branch ligations. Intraoperative Doppler assessment confirmed restoration of adequate venous flow.

#### CONCLUSION

LRV transposition provides definitive anatomic correction and durable decompression of the renal vein, making it a suitable option in selected patients with severe or combined manifestations of NCS. Simultaneous treatment of associated varicocele may further improve clinical outcomes and reduce symptom persistence. This case emphasizes the importance of individualized treatment strategies for NCS and supports the role of surgical management in patients with symptomatic NCS accompanied by significant gonadal vein reflux.

#### KEYWORDS

nutcracker syndrome, varicocele, open surgery, renal vein transposition, case report

**Figure:**
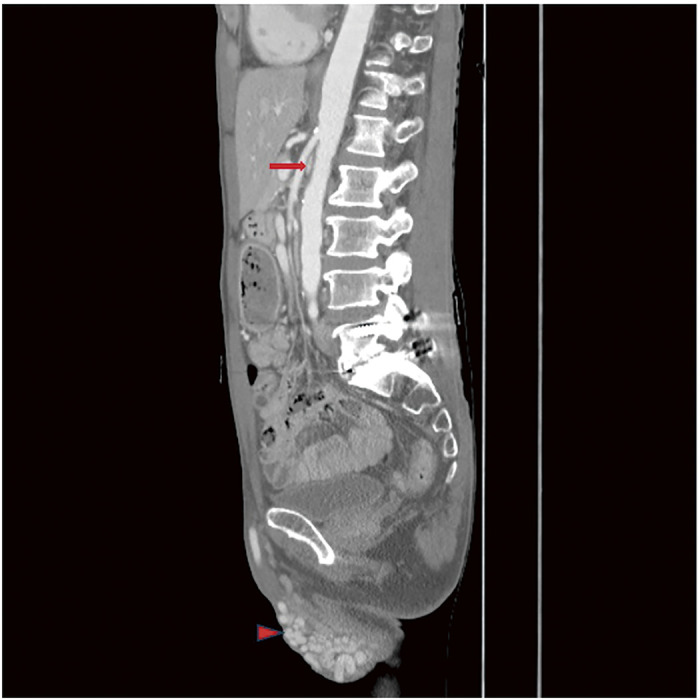


**Figure:**
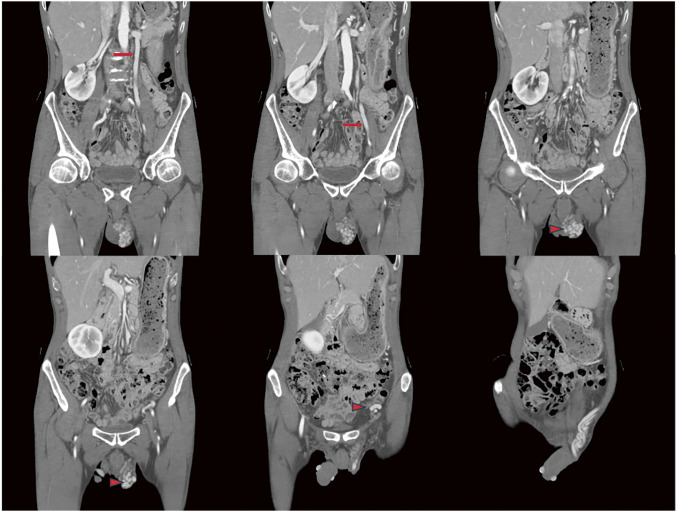


### EP-8 About High-Sensitivity C-Reactive Protein in Laboratory Examinations

Lee Chan Jang

Department of Surgery, College of Medicine, Chungbuk National University, Korea

#### INTRODUCTION

We have long thought that atherosclerosis has an inflammatory component and is associated with cardiovascular risk. But atherosclerosis is not a potent inflammatory process compared to infection. The risk factors for atherosclerosis include age, sex, diabetes, kidney function, cholesterol, an inflammatory process without infection, smoking, hypertension, lifestyle habits, and so on. We would like to know the level of high-sensitivity C-reactive protein (hs-CRP) according to the risk factors identified by laboratory examinations.

#### METHODS

A total of 666732 records of hs-CRP level and 54579 of low-density lipoprotein cholesterol (LDL-cholesterol) were reviewed. Data were collected between February 25, 2004, and November 15, 2019, at Chungbuk National University Hospital in Korea. Data were analyzed according to age, sex, serum creatinine, blood sugar, and LDL-cholesterol.

#### RESULT

The mean hs-CRP level increased with age in all age groups, and men had higher levels than women. The mean hs-CRP levels were higher for abnormal serum creatinine than that for abnormal blood sugar in all age groups. On the basis of age 60, factors that increased hs-CRP included serum creatinine (4.43 vs. 6.01), blood sugar (3.7 vs. 4.51), and increasing age (1.55 vs. 2.73). LDL-cholesterol showed a slightly negative correlation with hs-CRPs.

#### CONCLUSION

The data used in this study primarily come from patients who visited our hospital and are not representative of the global population. As I mainly used local data, each hospital needs to check its own data.

#### KEYWORDS

hs-CRP, serum creatinine, blood sugar, age, LDL-cholesterol

### EP-9 Treatment for Infected Aortic Aneurysm of Post-EVAR Patient with Long Peritoneal Dialysis History

Minsang Song

Dongrae Bongseng Hospital, Korea

#### INTRODUCTION

The gold standard treatment for aortic vascular graft and endograft infection is radical resection of the infected graft with vascular reconstruction. However, for patients who cannot undergo surgery due to various complications, conservative treatment is the only option.

#### METHODS

We present a case of an 81-year-old man with an infected aortic aneurysm of post-endovascular aneurysm repair (EVAR) patent who was successfully treated with only palliative treatment. This patient visited the hospital complaining of lower back pain that had started a month ago and had undergone EVAR surgery for abdominal aortic aneurysm 44 months ago. The patient underwent emergency surgery due to pain and fever that were not controlled despite 3 days of conservative treatment, including intravenous fluids and antibiotics. Due to a history of peritoneal dialysis for the past 10 years, the patient had severe intra-abdominal adhesions and severe inflammatory changes around the abdominal aortic aneurysm, making access difficult, and difficulty in maintaining blood pressure during surgery; thus, only drainage and irrigation were possible.

#### RESULT

The surgery was completed after inserting a drainage tube around the aneurysm and into the pelvis, and the patient recovered after 6 weeks of intravenous antibiotic treatment. The patient has been taking antibiotics continuously since discharge and has survived for more than 2 years.

#### CONCLUSION

Even though only palliative treatment was possible in this case, this treatment may be an option for patients with poor general condition.

### EP-10 Gonadal Vein Embolization for Pelvic Congestion Syndrome

Minhyung Lee, Hyunjeong Rhy, Yongman Park, Junkee Park, Sinseok Yang, Yangjin Park

Samsung Medical Center, Sungkyunkwan University, Korea

#### INTRODUCTION

Pelvic congestion syndrome (PCS) is caused by pelvic venous insufficiency and is a recognized cause of chronic pelvic pain. Gonadal vein embolization (GVE) is a minimally invasive treatment option. This study evaluated the clinical effectiveness and safety of GVE in patients with PCS.

#### METHODS

We retrospectively reviewed 35 patients treated between 2014 and 2024. All patients demonstrated gonadal vein reflux on duplex ultrasonography and underwent catheter-based venography. Clinical outcomes were assessed approximately 1 month after the procedure based on patient-reported symptom improvement.

#### RESULT

A total of 40 gonadal veins were embolized, with a 100% technical success rate. Follow-up duplex ultrasound at 1 month confirmed sustained occlusion in all available cases (38/38). Pelvic symptoms improved in 19 of 21 patients (90.5%); the remaining 2 patients with unchanged symptoms were presumed to have pain of nonvascular etiology. Regarding leg heaviness or cramping, all patients without lower extremity (L/E) reflux (6/6) improved after GVE alone. Among 18 patients with L/E reflux, those who underwent concurrent varicose vein surgery showed a 100% improvement rate (10/10), whereas those treated with GVE alone reported 62.5% improvement (5/8). Only 2 minor complications occurred.

#### CONCLUSION

GVE is a safe and effective treatment for pelvic symptoms in PCS, with excellent technical success. Careful patient selection is required, particularly in those presenting with atypical or nonpelvic symptoms.

**Figure:**
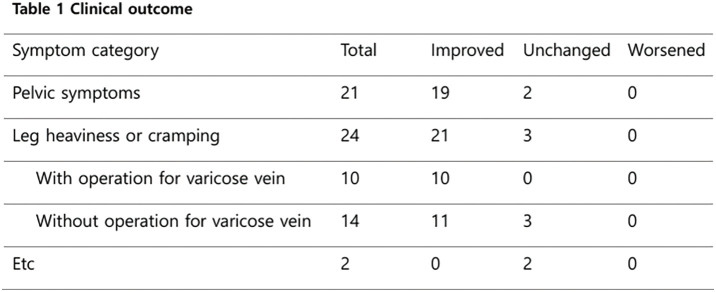


**Figure:**
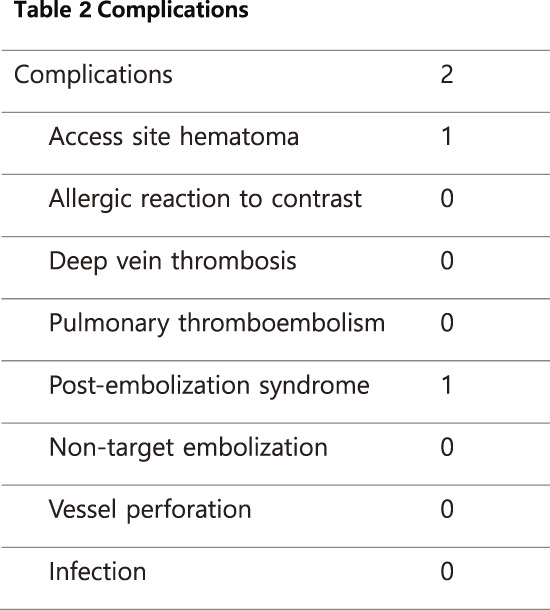


### EP-11 Early and Mid-Term Outcomes of Endovascular Aneurysm Repair Using Low-Profile Endografts

Minhyung Lee, Hyungjeong Ryu, Yongman Park, Junkee Park, Sinseok Yang, Yangjin Park

Samsung Medical Center, Sungkyunkwan University, Korea

#### INTRODUCTION

Endovascular aneurysm repair (EVAR) in patients with small access vessels or complex aortic necks remains clinically challenging. Low-profile endografts were developed to overcome these anatomical constraints. This study investigated the early and mid-term clinical outcomes of EVAR performed using low-profile devices.

#### METHODS

We retrospectively reviewed 48 patients who underwent EVAR with low-profile endografts (Incraft, n = 34; Minos, n = 14) between 2019 and 2025. The primary endpoint was early clinical outcome (technical success and 30-day complications), and secondary endpoints included mid-term clinical success and reintervention-free survival. Median follow-up was 23.1 months [interquartile range, IQR 4.3–38.4].

#### RESULT

Planned adjuvant procedures (iliac artery PTA, 16.7%; CFAE, 4.2%) were performed in 18.8% of cases to facilitate delivery. Technical success was 93.8% (45/48), with zero 30-day mortality. One unplanned CFA dissection (2.1%) required surgical repair. Kaplan–Meier estimates showed a clinical success rate of 83.3% at 40 months. The reintervention rate was 14.6% (7/48), driven by type II (n = 5), type Ia (n = 1), and type Ib (n = 1) endoleaks. One patient (2.1%) underwent late open conversion due to type II endoleak-induced sac growth. No instances of migration, limb occlusion, or abdominal aortic aneurysm-related mortality occurred.

#### CONCLUSION

EVAR with low-profile endografts is safe and effective for patients with challenging anatomy. These devices demonstrated high technical success and mid-term durability.

**Figure:**
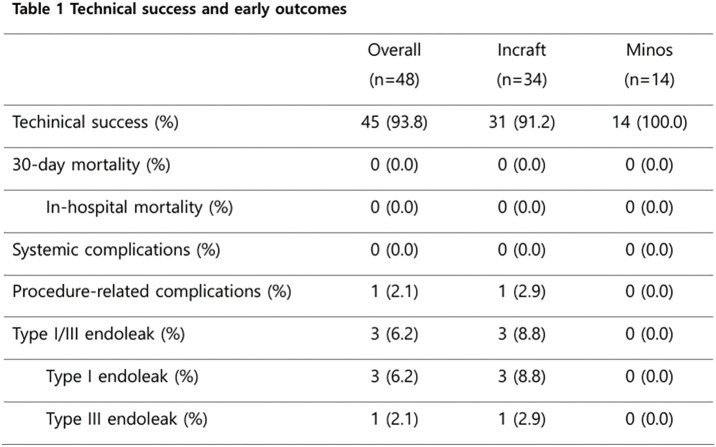


**Figure:**
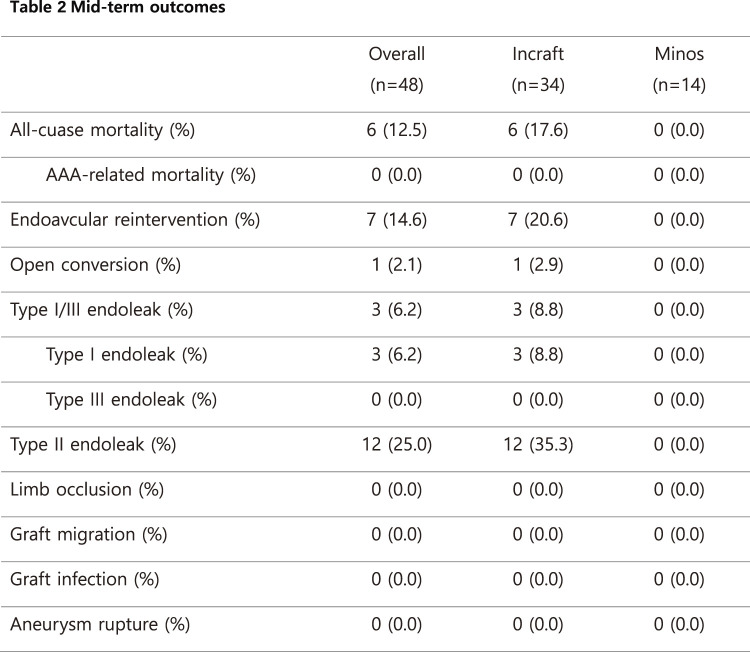


### EP-12 Correlation between Doppler Ultrasound Parameters and ABI in Revascularized Lower Extremity PAD

Jinmyeong Yoon

Department of Surgery, Seoul National University Bundang Hospital, Seongnam 13620, Republic of Korea

#### INTRODUCTION

Noninvasive arterial tests are essential in peripheral arterial disease (PAD), but the ankle-brachial index (ABI) may not accurately assess the effects of revascularization, particularly in severe calcification. Therefore, new parameters that can be derived from Doppler ultrasonography (DUS) have been proposed as an alternative. This study evaluated the accuracy of DUS-derived parameters in patients undergoing PAD revascularization.

#### METHODS

In this single-center retrospective study (January 2022–December 2025), patients undergoing open surgery or percutaneous transluminal angioplasty for lower extremity PAD were included. DUS parameters—acceleration time (AT), acceleration indices (AccI, AccI2), peak systolic velocity (PSV), total velocity (T), and summated total velocity (Tsum)—were collected pre- and post-revascularization. The correlation coefficients between these parameters and ABI were calculated, and receiver operating characteristic (ROC) curves were plotted using the commonly accepted ABI normal range of 0.9 to estimate the optimal cutoffs.

#### RESULT

A total of 42 patients and 48 limbs who had both pre- and post-revascularization data were included. AT, AccI, AccI2, T, Tsum, and PSV significantly correlated with ABI (r = 0.763, 0.740, 0.774, 0.824, 0.840, and 0.719). The ROC-derived cutoffs were AT = 0.138 s, T = 44, AccI2 = 440, and Tsum = 140. Tsum showed the strongest correlation with ABI, whereas AccI2 correlated better than AccI and T better than PSV.

#### CONCLUSION

Tsum showed the strongest correlation with ABI in reflecting improved lower-extremity perfusion. Further studies evaluating wound healing are needed to confirm their prognostic value.

### EP-13 A Case of a Soft Tissue Tumor Near the Greater Saphenous Vein Suspected to be a Venous Aneurysm

Keiko Urushino, Kenshi Yoshimura

The Department of Cardiovascular Surgery, Nakatsu Municipal Hospital, Oita, Japan

#### INTRODUCTION

The patient was a 75-year-old woman who had noticed a small lump on the medial aspect of her right lower leg for several years. Recently, it began enlarging and caused pain radiating to her toes upon palpation, prompting a visit to her primary care physician. Suspecting a venous disorder, she was referred to our department.

#### METHODS

A lower extremity ultrasound revealed a well-defined, solid tumor approximately 8 mm in diameter adjacent to the great saphenous vein. No blood flow was detected within the tumor lumen; it appeared as a tumor with abundant blood flow supplied by feeding vessels from the periphery. No reflux was observed throughout the entire length of the great saphenous vein. Initially, it was considered a venous aneurysm originating from the great saphenous vein. However, after additional imaging studies and consideration of epidemiological factors, an angioleiomyoma was deemed the most likely differential diagnosis.

#### RESULT

Due to the presence of pain, a simple excision of the tumor was performed under local anesthesia for both diagnostic and therapeutic purposes. Macroscopically, the excised tumor was a milky-white, glossy mass. Pathologic diagnosis revealed a benign soft tissue tumor composed of spindle-shaped cells. Immunohistochemically, many tumor cells and normal peripheral nervous tissue were positive for S100 protein and negative for desmin, α-smooth muscle actin, c-kit, and CD34, compatible with schwannoma.

#### CONCLUSION

Soft tissue tumors of the peripheral extremities, including neural tumors, angioleiomyoma, and so on can be encountered in venous practice. It is considered useful to have knowledge of the disease concepts as differential diagnoses.

#### KEYWORDS

venous aneurysm, soft tissue tumor, schwannoma, angioleiomyoma

**Figure:**
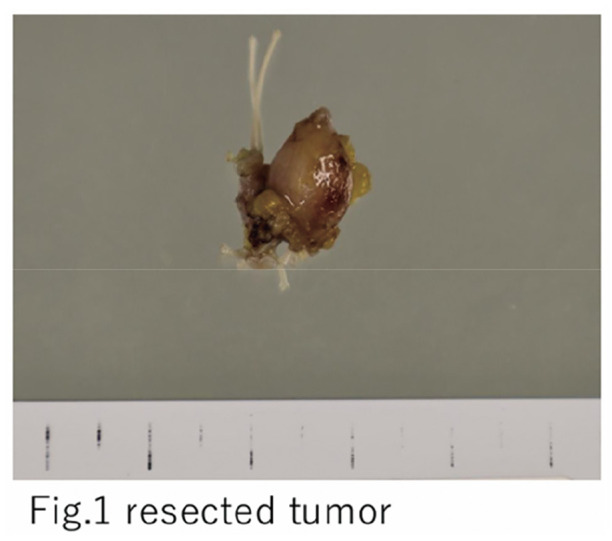


**Figure:**
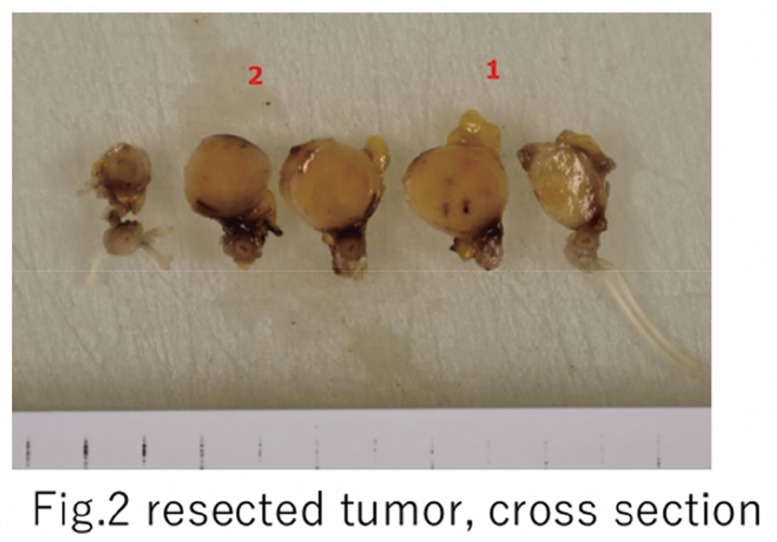


### EP-14 Extra-Anatomic Bypass Avoiding Infected Groin, Addressing Multiple Challenging Surgical Techniques

Kyokei Fuchizawa, MD, Shinsuke Kikuchi, MD, PhD, Izumi Fukii, MD, Hirofumi Jinno, MD, Takayuki Uramoto, MD, Keisuke Kamada, MD, Naoya Kuriyama, MD, Nobuyoshi Azuma MD, PhD

Department of Vascular Surgery, Asahikawa Medical University, Asahikawa, Japan

#### INTRODUCTION

In femoral arterial infections, placing prosthetic material or routing a bypass through the infected field is discouraged because of the risk of persistent or recurrent infection; therefore, extra-anatomic reconstruction that bypasses the infected groin has been advocated.

#### METHODS

An octogenarian man was emergently admitted with cardiogenic shock due to sustained ventricular tachycardia and underwent placement of an Impella™ device via the left common femoral artery (CFA). During device removal, an intimal injury occurred. Despite multiple repair procedures, including patch angioplasty, performed by another department, the lesion ultimately became infected and developed a pseudoaneurysm. The patient also had a 34-mm abdominal aortic aneurysm. Considering poor-quality great saphenous vein, excessive bypass length, and the need for future endovascular aneurysm repair access, a contralateral femoral vein graft was selected. A lateral extra-anatomic bypass was constructed using end-to-end anastomoses after transection and mobilization of the external iliac and superficial femoral arteries, creating a shorter and straighter reconstruction that avoided the infected CFA and provided a natural access route. The infected CFA was excised, and the deep femoral artery was ligated. The groin wound was managed by open drainage and subsequent skin grafting.

#### RESULT

At 6-month follow-up, the reconstruction remained patent without any complications.

#### CONCLUSION

This technique overcomes several procedural and anatomical limitations encountered in complex groin pathology. The present case is reported together with a review of the relevant literature.

#### KEYWORDS

extra-anatomic bypass, femoral arterial infections

### EP-15 Outcome of Explantation with In Situ Reconstruction for Aortic Graft Infection

Yong-Man Park

Division of Vascular Surgery, Department of Surgery, Samsung Medical Center, Sungkyunkwan University School of Medicine, Seoul, Korea

#### INTRODUCTION

Aortic graft infection is a life-threatening complication with 10%–40% mortality. Comparative outcomes of explantation with in situ reconstruction between endograft infection (EGI) and vascular graft infection (VGI) remain unclear. We compared outcomes of EGI versus VGI treated by explantation with in situ reconstruction.

#### METHODS

A retrospective single-center review included patients who had previously undergone endovascular aneurysm repair (EVAR) or open surgery for abdominal aortic aneurysm or aortoiliac occlusive disease and subsequently required explantation of an infected aortic graft with in situ reconstruction. Patients were classified into EGI (post-EVAR) and VGI (post-surgery). Primary endpoint was 30-day mortality; secondary endpoints included complications and 5-year survival.

#### RESULT

Twenty-one patients were analyzed (EGI, n = 13; VGI, n = 8). Median age was 71.0 years; EGI patients were older (77.0 vs. 67.5; P = 0.050). Cultures were positive in 85.7%. EGI required more proximal clamping (interrenal or above: 53.8% vs. 25%; P = 0.055). Thirty-day mortality was 19.0% (EGI 23.1% vs. VGI 12.5%; P = 1.000). Acute kidney injury (AKI) was numerically more frequent in EGI (38.5% vs. 25.0%; P = 0.656). At a median follow-up of 26.1 months, 5-year survival was 36.1% for EGI and 29.2% for VGI (P = 0.965).

#### CONCLUSION

Explantation with in situ reconstruction showed comparable outcomes between EGI and VGI. However, EGI required more proximal clamping due to suprarenal fixation, potentially contributing to higher AKI.

**Figure:**
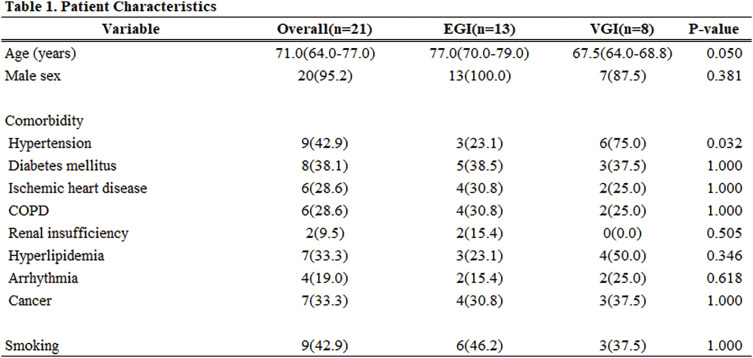


**Figure:**
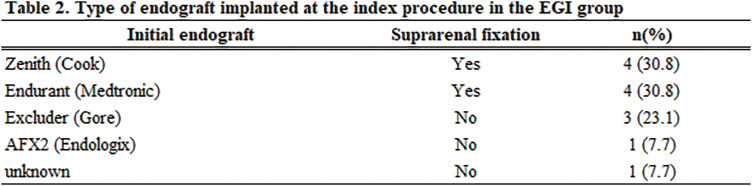


**Figure:**
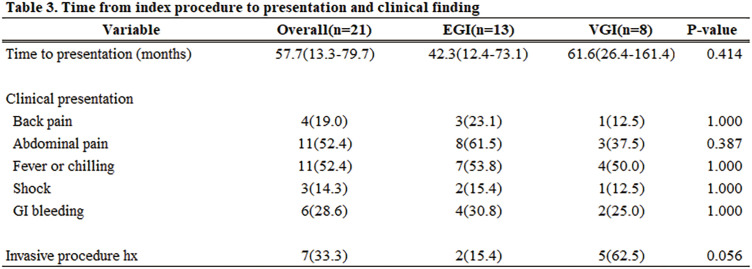


**Figure:**
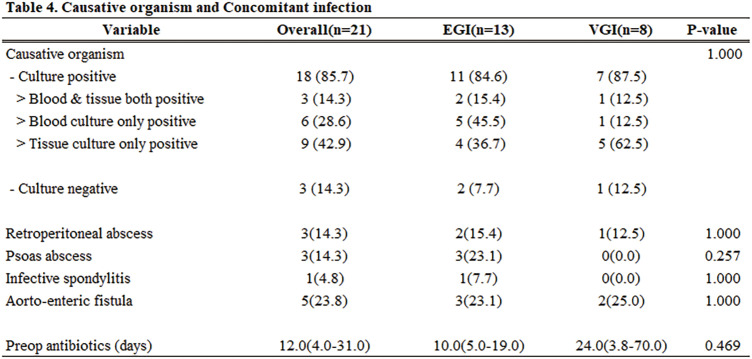


**Figure:**
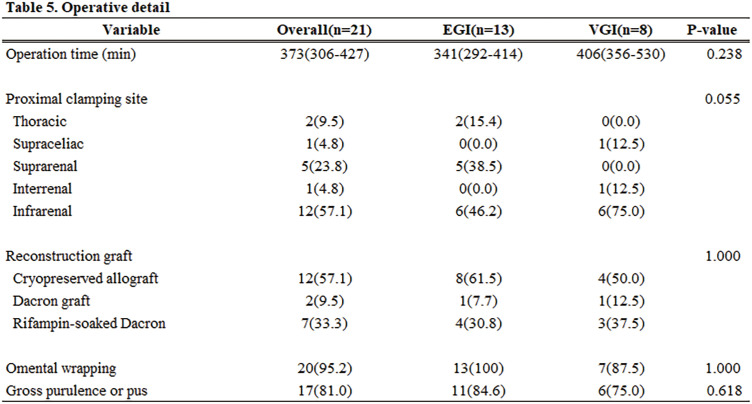


**Figure:**
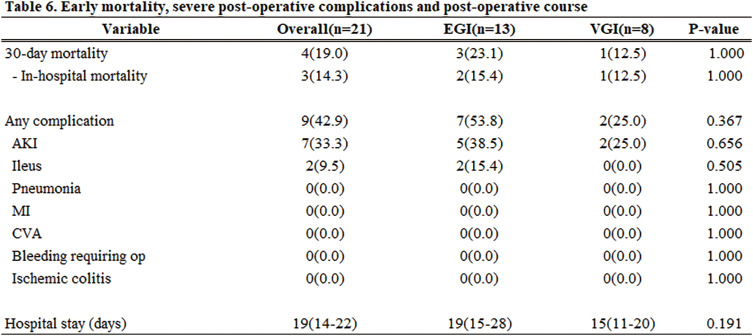


**Figure:**
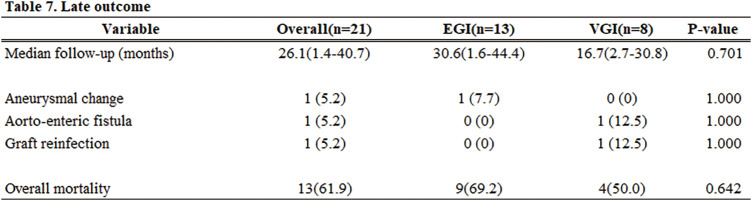


**Figure:**
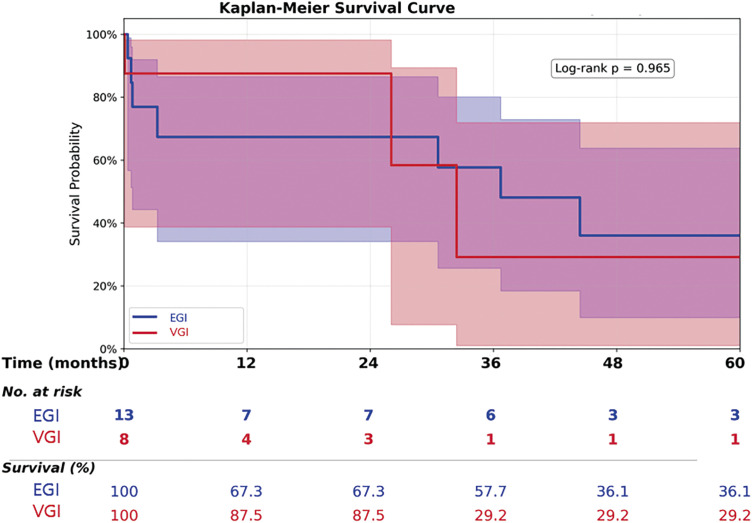


### EP-16 Severe Non-Ischemic Wrist Pain Leading to Arteriovenous Fistula Ligation: A Case Report

Suyong Lee, Kyunglim Koo, Daehwan Kim

The Better Way Clinic, Korea

#### INTRODUCTION

Arteriovenous fistula (AVF) ligation is usually reserved for ischemia, infection, or risk of rupture. Pain alone is rarely considered an indication for discontinuing a functional AVF in the absence of ischemia. However, access-related pain can significantly impair dialysis tolerance and quality of life. We report a case in which severe non-ischemic wrist pain led to AVF ligation with symptom resolution after relocation of vascular access.

#### METHODS

A 55-year-old man with a nearly 40-year history of hemodialysis was referred for severe left wrist pain associated with a long-standing AVF. He had undergone multiple surgical and endovascular procedures at outside hospitals without durable relief and requested access relocation because the pain had become intolerable during dialysis. He had a patent left radiocephalic AVF created in 1986. The pain occurred mainly during dialysis and required analgesic use. On examination, the left wrist was enlarged with aneurysmal AVF dilation. There were no signs of ischemia, and hand perfusion and motor function were preserved. A right brachio-upper basilic arteriovenous graft was created on July 8, 2025. After dialysis was shifted to the new access, the wrist pain resolved immediately.

#### CONCLUSION

This case highlights severe non-ischemic pain as a rare but valid indication for AVF ligation. When pain is clearly access-related, refractory, and disabling, relocation of vascular access may be justified to improve quality of life.

### EP-17 Hypothenar Hammer Syndrome in a Transportation Worker: A Case Report

Eol Choi

Division of Vascular Surgery, Department of Surgery, Soonchunhyang University College of Medicine and Soonchunhyang University Bucheon Hospital, Bucheon, Republic of Korea

#### INTRODUCTION

Hypothenar hammer syndrome (HHS) is a rare cause of ulnar artery injury due to repetitive hypothenar trauma and may result in ulnar-sided digital ischemia.

#### METHODS

A 63-year-old right-handed transportation worker presented with right hypothenar pain and swelling that began 1 month before presentation. He had hypertension, diabetes mellitus, and a smoking history, and complained of coldness, sensory decrease, and weakness of the fourth and fifth digits. Examination revealed hypothenar tenderness, palpable radial pulse, absent ulnar pulse, and a negative Allen’s test indicating insufficient collateral circulation. Doppler ultrasonography and computed tomography angiography demonstrated segmental occlusion with aneurysmal change of the distal ulnar artery anterior to the hook of the hamate (Fig. 1). No embolic or diffuse atherosclerotic source was identified, and HHS was suspected.

#### RESULT

Surgery revealed a thrombosed aneurysmal segment at the ulnar artery bifurcation. Resection and reconstruction using a Y-shaped autologous superficial vein graft were performed (Fig. 2). After postoperative immobilization, the patient returned to work at 3 weeks with near-normal motor recovery and resolution of pain, coldness, and sensory disturbance.

#### CONCLUSION

HHS should be considered in patients with ulnar-sided hand symptoms and repetitive occupational hypothenar stress. Surgical reconstruction can provide excellent functional recovery when collateral circulation is inadequate.

**Figure:**
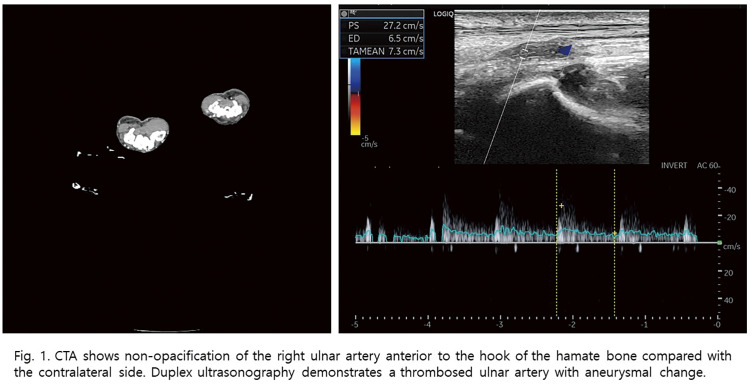


**Figure:**
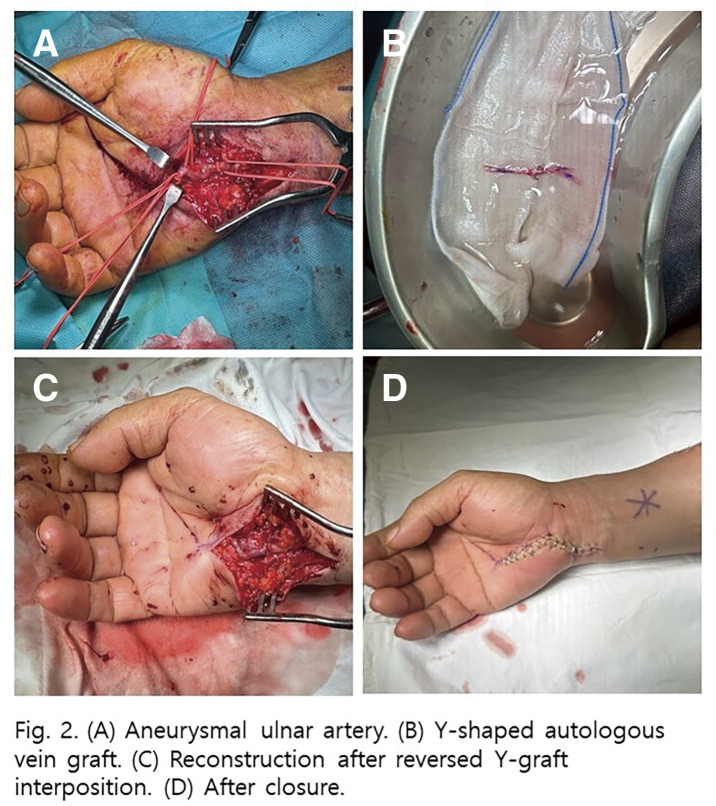


### EP-18 Efficacy of Reverse Slider Technique and EndoSuture Aneurysm Repair in Hostile Neck Abdominal Aortic

Seong Jun Lim, MD, PhD, Su Ji Oh, MD, Hae Lee, MD, Myeong Su Kim, MD, Suk-Won Song, MD, PhD

Department of Thoracic and Cardiovascular Surgery, Ewha Womans University Medical Center, Ewha Womans University College of Medicine, Korea

#### INTRODUCTION

Hostile neck anatomy in endovascular aneurysm repair (EVAR) is associated with proximal sealing failure and type Ia endoleak. We evaluated the outcomes of combining the reverse slider technique with EndoSuture Aneurysm Repair (ESAR) in patients with hostile neck abdominal aortic aneurysms (AAAs).

#### METHODS

We retrospectively reviewed patients who underwent EVAR with adjunctive reverse slider technique and ESAR at our institution between 2023 and 2025. Primary outcomes were absence of type Ia endoleak and aneurysm sac regression or stability. Secondary outcomes included technical success, migration, proximal reintervention-free survival, and perioperative complications. Follow-up imaging with computed tomography angiography (CTA) was performed at 30 days, 6–12 months, and annually.

#### RESULT

A total of 42 patients were included. Technical success was achieved in 100%. No type Ia endoleak was observed intraoperatively or during follow-up. Sac regression occurred in 48% of patients, while enlargement was rare. Endograft migration ≥10 mm occurred in 2 patients (4.9%), and aneurysm-related reintervention was required in 2 patients (4.9%). One patient (2.4%) died during follow-up, but most survived free of aneurysm-related events. Complications were uncommon.

#### CONCLUSION

The combined reverse slider technique and ESAR achieved excellent proximal sealing with no type Ia endoleak, low migration and reintervention rates, and favorable sac behavior. This strategy may expand the applicability of EVAR in challenging anatomies.

### EP-19 Outcomes of Open Conversion for Failed EVAR

Seong Jun Lim, MD, PhD, Su Ji Oh, MD, Hae Lee, MD, Myeong Su Kim, MD, Suk-Won Song, MD, PhD

Department of Thoracic and Cardiovascular Surgery, Ewha Womans University Medical Center, Ewha Womans University College of Medicine, Korea

#### INTRODUCTION

This study aimed to evaluate clinical characteristics, operative strategies, and outcomes of open surgical conversion after failed endovascular aneurysm repair (EVAR), with emphasis on procedural urgency and the reliability of preoperative computed tomography (CT) findings in patients with aneurysm sac enlargement.

#### METHODS

This retrospective multicenter study included 94 patients who underwent open conversion after failed EVAR between 2017 and 2025. Patients were classified as elective or urgent/emergent. Perioperative and long-term outcomes were analyzed. In patients with sac enlargement, concordance between CT-based endoleak classification and intraoperative findings was assessed.

#### RESULT

Of the 94 patients, 39 (41.5%) underwent elective and 55 (58.5%) urgent/emergent conversion. Partial endograft explantation was the most common strategy. Early postoperative complications were more frequent in the urgent/emergent group (29.1% vs. 10.3%). Overall complication and abdominal aortic aneurysm–related mortality rates were 25.5% and 7.4%, respectively. Survival did not differ by urgency, but elective conversion was associated with superior complication-free survival. Among 53 patients analyzed for sac enlargement, 54.3% of those diagnosed with type II or V endoleak on CT were found to have type I or III endoleaks intraoperatively.

#### CONCLUSION

Open conversion after failed EVAR is feasible with acceptable outcomes when appropriately timed. In patients with progressive sac enlargement, CT alone may underestimate clinically significant endoleaks, supporting earlier consideration of open conversion.

### EP-20 Iatrogenic Arteriovenous Fistula after Arterial Line Injection

Yujin Kwon

Seoul Medical Center, Korea

#### INTRODUCTION

Arterial line insertion is a common procedure in patients treated in the intensive care unit (ICU) for continuous hemodynamic monitoring. Although it is generally safe, rare vascular complications can occur. We report a case of an iatrogenic arteriovenous fistula formed between the radial artery and an adjacent vein following arterial line insertion.

#### METHODS

A patient underwent a craniotomy for a frontotemporoparietal intracerebral hemorrhage. During the subsequent ICU stay, a radial arterial catheter was inserted into the right radial artery for monitoring. After discharge, the patient noted progressive, tortuous enlargement of a superficial vein at the wrist. On physical examination, a palpable thrill was detected over the previous arterial puncture site. Ultrasonography revealed an arteriovenous fistula (AVF) between the radial artery and an adjacent vein, with arterialized flow in the cephalic vein.

#### RESULT

The patient underwent surgical closure of the AVF and vein excision, and complete resolution of the thrill was confirmed with normalized arterial flow.

#### CONCLUSION

Although rare, iatrogenic AVF is a significant complication of arterial cannulation. A high index of clinical suspicion is required for patients presenting with localized swelling or a thrill at the puncture site. Early surgical management remains the definitive treatment to prevent long-term sequelae.

### EP-21 Rare Cause of Lower Extremity Venous Obstruction by Non-Vascular Cystic Disease

Jaehoon Lee, Kihyuk Park

Daegu Catholic Medical University Hospital, Korea

#### INTRODUCTION

Rarely, cystic disease such as adventitial cystic disease (ACD) causes venous obstruction. According to an extensive literature review, only 53 cases have been reported to date. Cysts usually occur in blood vessels around the major joints, and patients complain of severe swelling, tenderness, and pain. Synovial cysts originating from joints also extremely rarely compress the femoral vein with similar symptoms. We have experienced 7 cases of cystic disease in lower extremities: 3 in the right legs, 4 in the common femoral vein, 2 in the external iliac vein, 1 in the popliteal vein.

#### RESULT

All patients were the visited vascular clinic with a swollen leg to rule out deep vein thrombosis (DVT) and later diagnosed as venous adventitial cystic disease after imaging tests. Five patients underwent surgical treatment, and 2 underwent conservative treatment with medical therapy. All surgical patients had successfully improved leg swelling after complete cyst removal. Intraoperative venogram was done to confirm vein patency. One patient had venous thrombosis before surgery during the preoperative admission period and underwent intraoperative thrombectomy with Angiojet catheter.

#### CONCLUSION

ACD and large synovial cysts could be misunderstood as DVT. Computed tomography or ultrasonogram test is enough for differentiation. Thrombosis occurs for this outflow obstruction status. Surgical removal of gelatinous cyst could offer definite treatment.

### EP-22 Bailout Chimney EVAR for Persistent Type Ia Endoleak in a Hostile Neck Abdominal Aortic Aneurysm

Hyung-jin Cho, Mi-hyeong Kim, Jisum Moon, Junsueng Kim, Jeong-kye Hwang

Division of Vascular and Transplant Surgery, Department of Surgery, Eunpyeong St. Mary’s Hospital, College of Medicine, The Catholic University of Korea, Seoul, Korea

#### INTRODUCTION

Endovascular aneurysm repair (EVAR) in hostile neck anatomy is associated with an increased risk of type Ia endoleak. Although EndoAnchors may improve proximal fixation, additional bailout strategies are sometimes required.

#### METHODS

A 92-year-old woman was referred for treatment of an infrarenal abdominal aortic aneurysm with a maximal diameter of 56 mm and severe proximal neck angulation of 79°. EVAR with adjunctive techniques, including EndoAnchor placement, was planned due to the hostile neck anatomy. During the procedure, a reverse slider technique was used, advancing the main body graft to partially cover the left renal artery. However, a persistent type Ia endoleak was observed. Repeated molding ballooning and deployment of approximately 10 EndoAnchors failed to achieve adequate proximal sealing. As a bailout procedure, a single renal chimney was performed using an aortic cuff and a covered stent (Covera Plus). Completion angiography demonstrated a marked reduction in type Ia endoleak flow.

#### RESULT

The procedure was concluded with close surveillance. Follow-up computed tomography showed no significant endoleak except for a suspected type II endoleak, and conservative management was chosen.

#### CONCLUSION

This case demonstrates that bailout renal chimney EVAR can be an effective salvage option for persistent type Ia endoleak in severely angulated hostile neck anatomy when EndoAnchor placement alone is insufficient.

**Figure:**
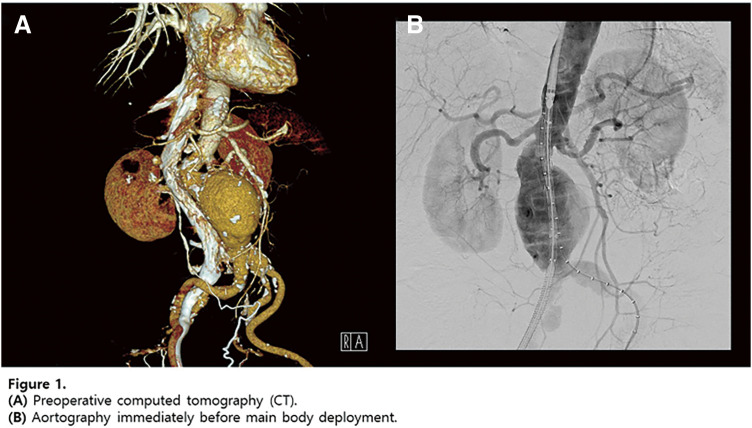


**Figure:**
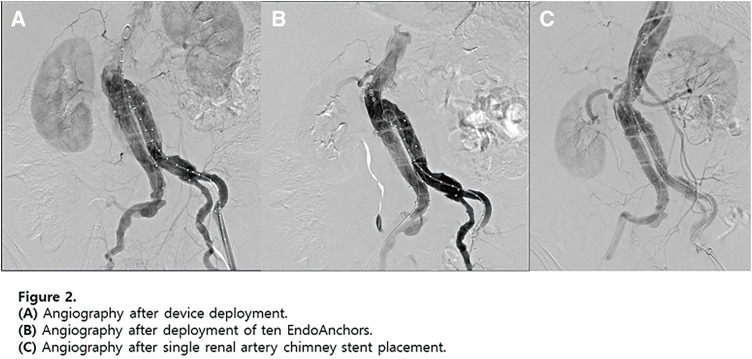


### EP-23 How Can Vascular Surgeons Contribute to Hemodialysis Access Care in Africa?

Kwan Tae Park

St. Peter’s General Hospital, Korea

#### INTRODUCTION

End-stage renal disease is rapidly increasing across Africa, while access to durable hemodialysis (HD) access remains severely limited. A lack of trained vascular surgeons, inadequate interventional facilities, and prolonged use of tunneled or temporary catheters contribute to poor access patency and high complication rates.

#### METHODS

We conducted repeated surgical missions to major tertiary hospitals in Africa, focusing on HD access surgery, access revision, and selective endovascular interventions. All procedures were performed in collaboration with local medical staff, with an emphasis on education and skill transfer rather than one-time service delivery.

#### RESULT

Over 7 visits, a total of 238 HD access surgeries, 25 endovascular interventions, and 31 kidney transplant–related procedures were performed. The scope of surgery expanded progressively with each visit, reflecting improved local collaboration and growing surgical capacity. Despite severe resource limitations, complex access revisions and flow-reduction procedures were successfully completed.

#### CONCLUSION

Sustainable improvement of HD access care in Africa requires repeated engagement, training-based collaboration, and leadership from countries with advanced HD access expertise. Korea and Japan, with globally recognized experience in HD access surgery, are well positioned to take a leading role in building long-term capacity across Africa.

#### KEYWORDS

hemodialysis access, arteriovenous fistula, low-resource settings, Medical volunteerism, Africa

### EP-24 From Startup to Standard: Analysis of Access-Related Complications in Our First 100 EVAR Cases

Hyung-jin Cho, Mi-hyeong Kim, Jisum Moon, Junseung Kim, Jeong-kye Hwang

Division of Vascular and Transplant Surgery, Department of Surgery, Eunpyeong St. Mary’s Hospital, College of Medicine, The Catholic University of Korea, Seoul, Korea

#### INTRODUCTION

Sheath-associated access complications during endovascular procedures have been reported to be related to multiple patient- and device-related factors, including body habitus and arterial characteristics. However, these associations may vary according to institutional experience. We revisited access-related events during the early experience of a newly established center.

#### METHODS

We retrospectively analyzed consecutive endovascular procedures requiring bilateral femoral access performed at Eunpyeong St. Mary’s Hospital between July 2019 and January 2026. Access-related events were defined as predefined adverse outcomes occurring at either access site. Baseline characteristics and procedural variables were evaluated. Univariate and multivariate logistic regression analyses were performed to identify factors associated with access-related events.

#### RESULT

Among 88 patients, 17 (19.3%) experienced access-related events. In univariate analysis, events were associated with longer operation time (P = 0.017), increased blood loss (P = 0.044), lower American Society of Anesthesiologists (ASA) class (P = 0.036), and antiplatelet therapy (P = 0.003). In multivariate analysis, antiplatelet therapy (odds ratio [OR] 0.19; 95% confidence interval [CI] 0.05–0.66; P = 0.009) and ASA class (OR 0.33; 95% CI 0.11–0.95; P = 0.041) remained independently associated with access-related events.

#### CONCLUSION

Access-related events were more closely related to baseline patient risk and procedural complexity than to sheath size itself, highlighting the importance of context-specific risk assessment.

**Table 1:** 

Characteristics	No. (%) (N = 88)
Age (years)	76.3 ± 7.7
Sex (male)	79 (89.8%)
BMI (kg/m^2^)	23.8 ± 3.3
Smoking history	
Never	51 (75.0%)
Current	16 (18.2%)
Ex-smoker	21 (23.9%)
Past Hx.	
Hypertension	66 (75.0%)
Diabetes mellitus	23 (26.1%)
Dyslipidemia	55 (62.5%)
Ischemic heart disease	65 (73.9%)
Cerebrovascular disease	21 (23.9%)
Peripheral artery disease	7 (8.0%)
Congestive heart failure	3 (3.4%)
COPD	9 (10.2%)
Chronic kidney disease	16 (18.2%)
End-stage renal disease	3 (3.4%)
Preoperative laboratory tests	
Preoperative hemoglobin (g/dL)	12.3 ± 1.9
Preoperative creatinine (mg/dL)	1.3 ± 1.1
Preoperative medication Hx.	
Antiplatelet therapy	59 (67.0%)
Anticoagulation therapy	8 (9.1%)
Operative-related factor	
Urgent procedure	4 (4.5%)
Operation time (min)	127.0 ± 61.2
Blood loss (mL)	124.3 ± 199.5

**Table 2:** 

Univariate analysis			
Variable	Complication (+) (N = 17)	Complication (−) (N = 71)	p Value
Age (years)	75.8 ± 8.2	76.4 ± 7.6	0.781
BMI (kg/m^2^)	22.7 ± 3.3	24.0 ± 3.2	0.164
Operation time (min)	158.9 ± 56.5	119.4 ± 60.2	**0.017**
Blood loss (mL)	259.4 ± 310.8	92.0 ± 147.9	**0.044**
ASA class	2.8 ± 0.4	3.1 ± 0.7	**0.036**
Antiplatelet therapy	6 (35.3%)	53 (55.2%)	**0.003**
Hypertension	10 (58.8%)	56 (58.3%)	0.118
Ischemic heart disease	7 (41.2%)	58 (60.4%)	0.355
Multivariate analysis			
Variable	OR	95% CI	p Value
Antiplatelet therapy	0.19	0.05–0.66	**0.009**
Operation time (min)	1.01	0.99–1.02	0.103
Blood loss (mL)	1.00	0.99–1.01	0.135
ASA class	0.33	0.11–0.95	**0.041**

### EP-25 Successful Hybrid Revascularization of Extensive Multi-Level Ilio-Femoro-Popliteal Artery Occlusion

Youngheun Shin, Doo-Ri Kim, In-Chul Nam

Department of General Surgery, Jeju National University Hospital, Jeju, Korea Department of Radiology, Jeju National University Hospital, Jeju, Korea

#### INTRODUCTION

Peripheral arterial disease (PAD) involving multilevel occlusions from the iliofemoral to the popliteal–tibial segments presents a significant therapeutic challenge. According to the TransAtlantic Inter-Society Consensus II (TASCII) guidelines, open surgical bypass has traditionally been the gold standard for such extensive TASC type D lesions. However, extensive bypass surgery carries a substantial risk of perioperative morbidity and mortality. While an “endovascular-first” strategy has become increasingly prevalent as a less invasive alternative, the failure rate of antegrade guidewire passage remains high for long, calcified, or multilevel chronic total occlusions (CTOs). In this context, a hybrid approach combining open surgery with endovascular therapy is proposed as a safe and effective alternative. We report a case of successful limb salvage using a sequential hybrid strategy in a patient with extensive ilio-femoro-popliteal occlusion.

#### METHODS

A 64-year-old patient presented with severe claudication. Computed tomography angiography revealed extensive multi-level occlusions: a 40-cm long chronic total occlusion from the left common iliac artery to the distal superficial femoral artery (SFA), combined with focal stenosis in the common femoral artery and segmental occlusion in the tibioperoneal (TP) trunk. Given the complexity and extent of the disease, a hybrid approach was planned. First, an open endarterectomy of the left femoral artery was performed to remove the calcified plaque and secure a reliable access point for intervention. Following endovascular reconstruction was attempted. After successful guidewire passage through the long-segment iliac and femoral CTO lesion, balloon angioplasty and stent insertion (common iliac artery, external iliac artery, and SFA) were performed. Additionally, balloon angioplasty was performed for the distal popliteal and TP trunk lesions. The final angiogram showed fully restored flow from the iliac artery down to the tibial vessels.

#### RESULT

At the 8-month follow-up, despite a minor re-intervention for focal restenosis, the patient demonstrated significant clinical improvement with an ankle-brachial index of 1.04/0.91 and limb salvage.

#### CONCLUSION

Our case demonstrates that a sequential hybrid strategy, combining open common femoral artery endarterectomy with extensive endovascular recanalization, serves as an effective and durable alternative to major bypass surgery for salvaging limbs in complex multi-level arterial occlusions.

**Figure:**
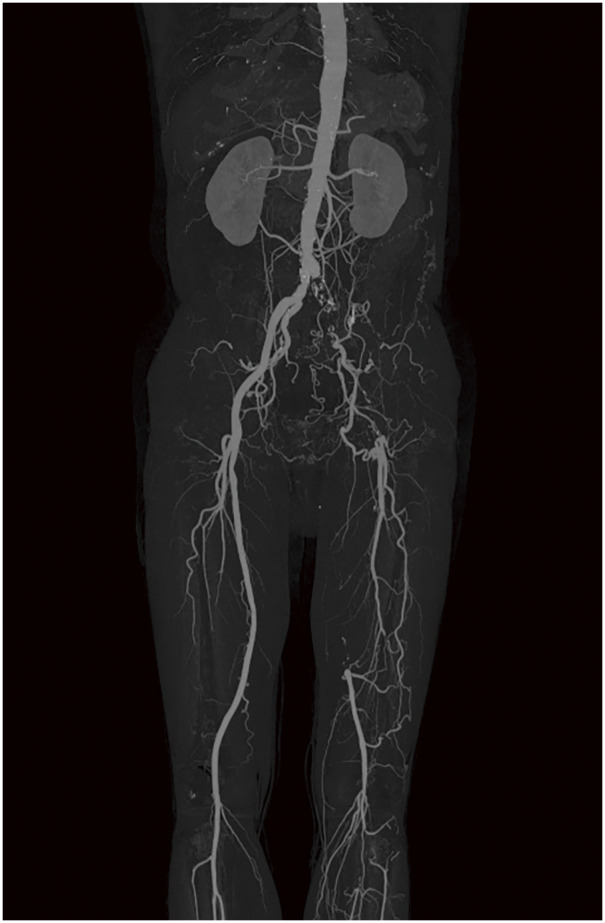


**Figure:**
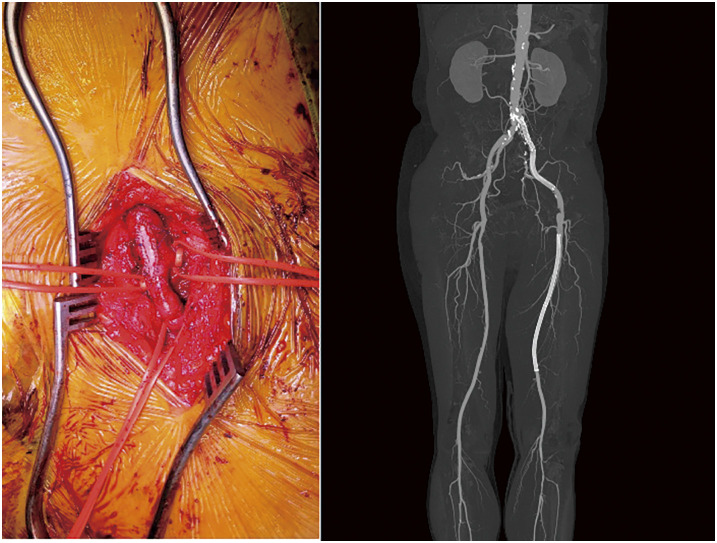


### EP-26 Accelerated Abdominal Aortic Aneurysm Expansion after Revascularization for Leriche Syndrome

Ju Yeon Choi, Youngjin Han

Department of Surgery, Armed Forces Capital Hospital, Seoungnam, Korea Department of Vascular Surgery, Asan Medical Center, Seoul, Korea

#### INTRODUCTION

In patients with Leriche syndrome and chronic aortoiliac occlusive disease, severely reduced distal perfusion may obscure the natural progression of a coexisting abdominal aortic aneurysm (AAA). Restoration of aortoiliac inflow can significantly alter aneurysmal hemodynamics.

#### METHODS

A patient presented with severe bilateral claudication. Ankle-brachial index was markedly reduced (right: 0.26; left: 0.37). Computed tomography angiography revealed Leriche syndrome with aortoiliac occlusive disease and a concomitant infrarenal AAA measuring 4.0 cm in diameter. In October 2024, aortoiliac kissing stent insertion combined with left common femoral artery endarterectomy was performed to relieve critical limb ischemia symptoms. The procedure successfully restored aortoiliac arterial flow.

#### RESULT

At 3-month follow-up, the aneurysm diameter increased to 4.3 cm. Despite continued surveillance, progressive aneurysmal expansion was observed, and the AAA reached 5.3 cm at 11 months after revascularization. Considering the rapid growth of the aneurysm following inflow restoration, stent explantation and open surgical repair were performed. The postoperative course was uneventful.

#### CONCLUSION

Restoration of aortoiliac inflow for severe claudication may unmask and accelerate aneurysmal progression in patients with coexisting AAA. Careful preprocedural assessment and close post-revascularization surveillance of coexisting AAA are crucial when planning aortoiliac revascularization.

### EP-27 MILLER-Type Banding with PTFE Strip for High-Flow Arteriovenous Fistula

Hyoung Tae Kim, Junmo Park, Sang Woo Kim

Vascular and Endovascular Surgical Clinic, Korea

#### INTRODUCTION

Various methods are known for repairing high-flow arteriovenous fistulas (AVFs). Each method has its own advantages and disadvantages, and it is difficult to definitively state that any 1 method is superior to the others. MILLER (minimally invasive limited ligation endoluminal-assisted revision) banding, a minimally invasive method with relatively predictable results, has recently become widely used. However, its major drawback is erosion of the suture material and the resulting complications.

#### METHODS

A 57-year-old man with a brachiocephalic AVF (BC AVF) showed high flow of 2326 mL/min on a 3-month sonogram. Follow-up sonogram revealed increased flow to 2711 mL/min, prompting the decision to perform flow reduction. We planned MILLER banding with a polytetrafluoroethylene (PTFE) strip. A PTFE graft was tailored into 1-cm-wide strips. A 1-cm incision was made just below the antecubital AVF, and the AVF was isolated. The PTFE strip was passed around the AVF. A 3-mm balloon catheter was positioned at the site of banding. While the balloon was inflated, the PTFE strip was tightened around the balloon and sutured with 6-0 polypropylene.

#### RESULT

Blood flow, which was 2711 mL/min preoperatively, decreased to 571 mL/min. After balloon removal, the final flow was 674 mL/min. Recovery was uneventful. One-month follow-up examination confirmed that blood flow was well maintained.

#### CONCLUSION

The MILLER-type banding using PTFE strips developed by the authors appears to be a promising method that maintains the advantages of the MILLER procedure while avoiding its major drawback, erosion caused by the suture material.

#### KEYWORDS

arteriovenous fistula, high flow, MILLER banding

**Figure:**
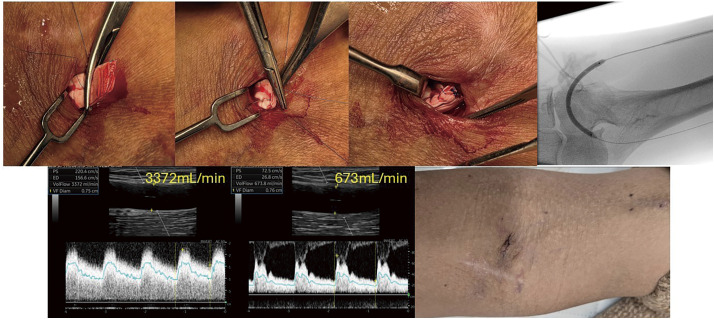


### EP-28 Emergency EVAR for Inflammatory or Infected AAAs: Two Cases without Reintervention

Togo Norimatsu, Nagaki Kiyohara

Department of Cardiovascular Surgery, Seirei Yokohama Hospital, Yokohama, Japan

#### INTRODUCTION

Endovascular stent-graft repair for abdominal aortic aneurysms associated with inflammatory or infectious pathology remains controversial. Nevertheless, in emergency situations, it may be considered as a bridging strategy when immediate open repair is undesirable.

#### METHODS

We report 2 cases of impending rupture of abdominal aortic aneurysms treated with emergency stent-graft repair under local anesthesia as bridging therapy. One patient had a pseudoaneurysm associated with Behçet’s disease, while the other had an infected aneurysm with rapid expansion. Although neither patient was hemodynamically unstable, both presented with symptomatic impending rupture requiring urgent intervention. Given the high surgical risk and underlying pathology, endovascular stent-graft repair was selected and successfully performed under local anesthesia in an emergency setting.

#### RESULT

Both procedures achieved immediate aneurysm exclusion without intraoperative complications. Postoperative courses were favorable. With appropriate adjunctive medical management, including immunosuppressive therapy for Behçet’s disease and prolonged antibiotic therapy for the infected aneurysm, no additional surgical or endovascular reintervention was required. During follow-up, the Behçet-related pseudoaneurysm remained stable for 24 months, and the infected aneurysm showed no evidence of recurrent infection or aneurysmal change for 10 months. Follow-up imaging demonstrated good stent-graft patency without endoleak or aneurysm-related complications.

#### CONCLUSION

Emergency stent-graft repair under local anesthesia can serve as an effective bridging strategy for impending rupture of abdominal aortic aneurysms, even in the presence of inflammatory or infectious conditions. In selected patients, appropriate perioperative medical therapy and careful follow-up may allow bridging treatment to result in sustained favorable outcomes without the need for reintervention.

#### KEYWORDS

inflammatory and infected aneurysm, EVAR, bridging therapy, impending rupture

### EP-29 Early Outcomes of Physician-Modified Endograft Implementation following Hands-On Workshop Training

Daisik Ko

Gachon University Gil Medical Center, Korea

#### INTRODUCTION

Physician-modified endograft (PMEG) is an effective endovascular option for complex abdominal aortic aneurysms (AAA), but its clinical adoption is limited by technical complexity and lack of structured training. After participating in a hands-on PMEG workshop in Hungary, we implemented PMEG in our clinical practice. This study reports early outcomes of our initial PMEG cases following workshop-based training.

#### METHODS

We retrospectively reviewed 3 consecutive patients who underwent PMEG for complex AAA. Two patients had infrarenal AAA with a dominant accessory renal artery preserved using a single fenestration. One patient had a juxtarenal AAA requiring fenestration of the superior mesenteric artery and both renal arteries. Preoperative planning was performed using contrast-enhanced computed tomography. Fenestrations were created using a punch-card technique and reinforced with Hungaro-rings. Technical success was defined as successful graft deployment with patent target vessels and no type I or III endoleak.

#### RESULT

PMEG was successfully deployed in all patients. All target vessels were cannulated and patent on completion of angiography. There were no type I or III endoleaks, perioperative mortality, major complications, or renal function deterioration. All patients were discharged without procedure-related complications.

#### CONCLUSION

PMEG can be safely introduced into clinical practice following structured hands-on training. Workshop-based education may shorten the learning curve and improve procedural safety.

**Figure:**
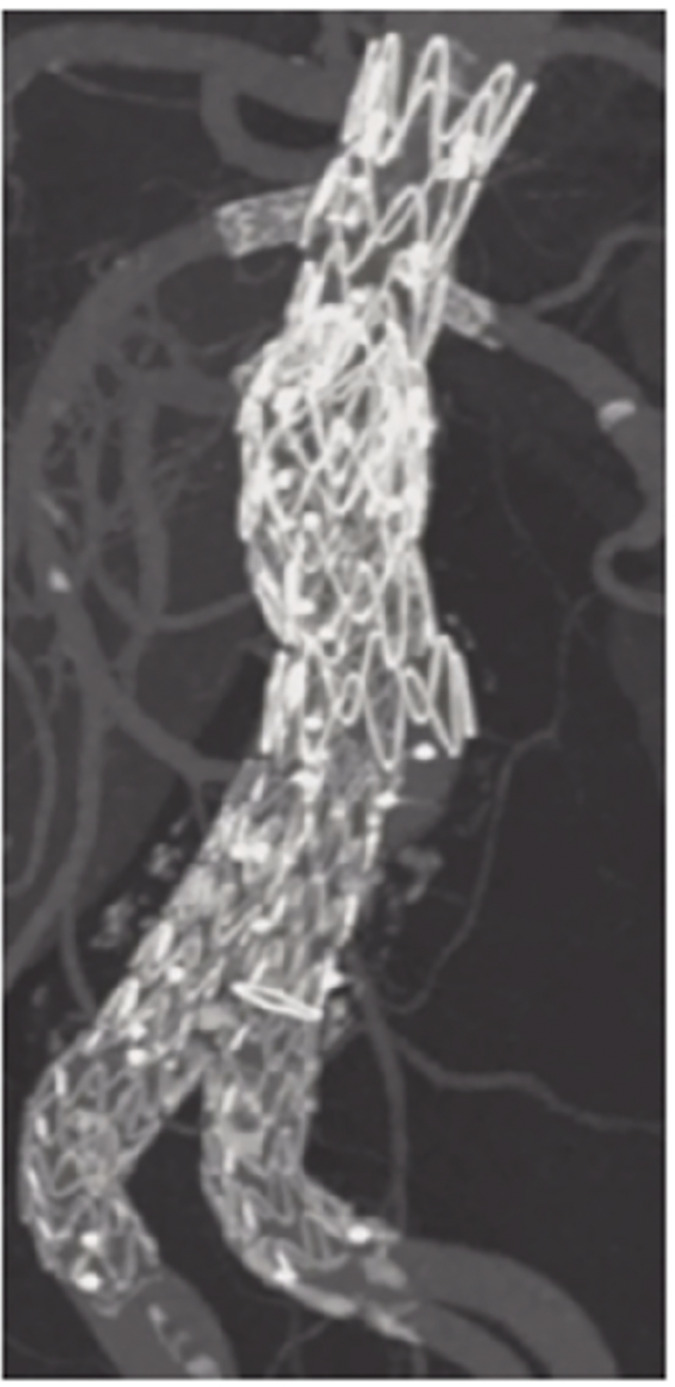


**Figure:**
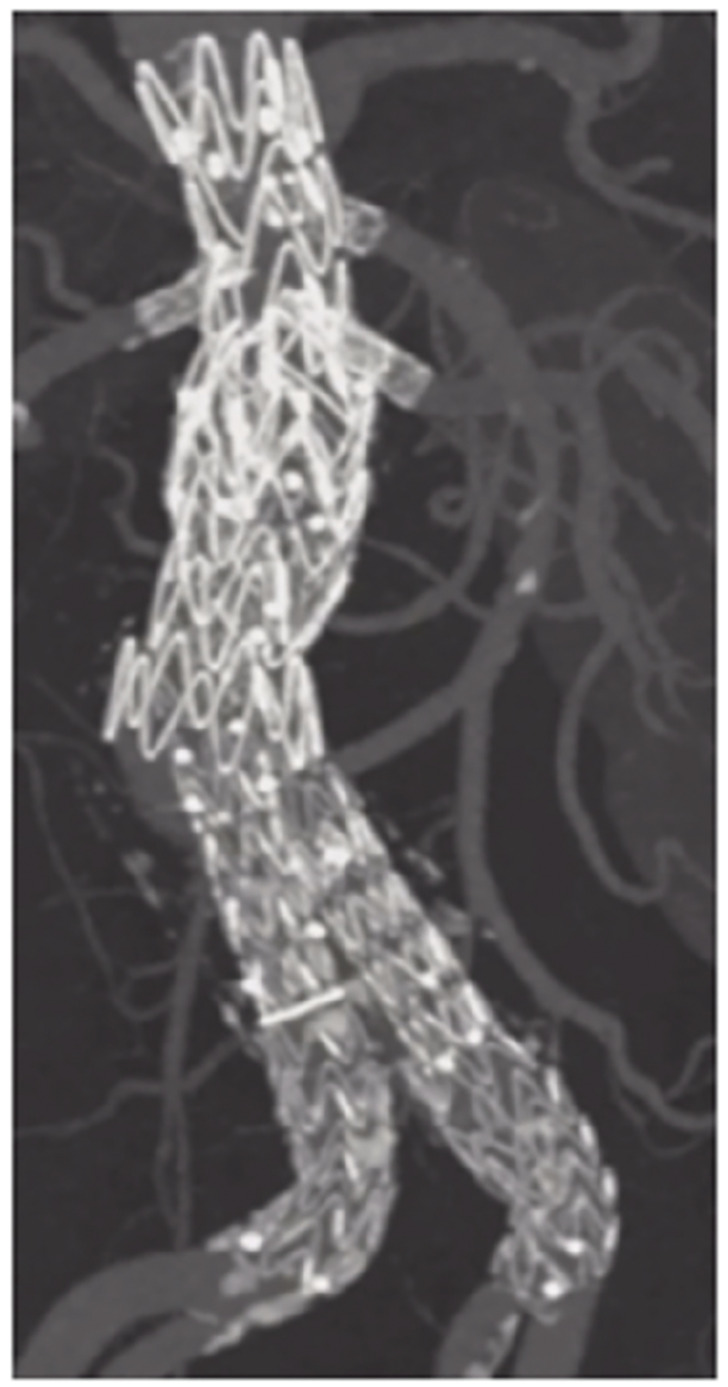


## Organizing Committee of the 14th Japan–Korea Joint Meeting for Vascular Surgery:

### Korean Society for Vascular Surgery

President: Yong-Pil Cho

Chair of Board: Sang-Su Lee

Secretary General: Jeongkye Hwang, Hyuk Jae Jung, Youngjin Han

Chairperson of Scientific Committee: Yang-Jin Park

### Japanese Society for Vascular Surgery

President: Nobuyoshi Azuma

Chairperson of the International Committee: Hiroyoshi Komai

